# Bioactive compounds from ShenFuShanYuRou decoction enhance Treg cell function against hemorrhagic shock injury via Stat1‐ and Gbp5‐dependent FOXP3 induction

**DOI:** 10.1002/ctm2.70047

**Published:** 2024-10-11

**Authors:** Qingxia Huang, Mingxia Wu, Lu Ding, Chen Guo, Yisa Wang, Zhuo Man, Hang Su, Jing Li, Jinjin Chen, Yao Yao, Zeyu Wang, Daqing Zhao, Linhua Zhao, Xiaolin Tong, Xiangyan Li

**Affiliations:** ^1^ Research Center of Traditional Chinese Medicine, College of Traditional Chinese Medicine Changchun University of Chinese Medicine Changchun Jilin China; ^2^ Northeast Asia Research Institute of Traditional Chinese Medicine, Key Laboratory of Active Substances and Biological Mechanisms of Ginseng Efficacy, Ministry of Education, Jilin Provincial Key Laboratory of Bio‐Macromolecules of Chinese Medicine Changchun University of Chinese Medicine Changchun Jilin China; ^3^ SCIEX China Beijing China; ^4^ Northeast Asia Research Institute of Traditional Chinese Medicine Changchun University of Chinese Medicine Changchun Jilin China

Dear Editor,

In this study, we unveiled the bioactive compounds and molecular mechanisms of ShenFuShanYuRou decoction (SFSY) against hemorrhagic shock/resuscitation (HS/R) injury via the promotion of regulatory T (Treg) cell function. Our work offers new therapeutic strategies for circumventing HS/R‐induced injury.

HS is a substantial global problem with more than 1.9 million deaths per year worldwide.^1^ While advances in resuscitation strategies have circumvented early mortality from HS, still ∼30% of patients experience multiple organ dysfunction (MOD).^2^ Treg cells play a vital role in maintaining innate immune homeostasis to foster tissue repair.^3^ Thus, multitarget regulation of Treg cell function offers a new therapeutic intervention to minimize HS/R injury. SFSY is a famous Traditional Chinese Medicine formula, widely used in the supplementary therapy of patients with shock in China. However, the bioactive compounds and molecular mechanisms of SFSY against HS/R injury have not yet been elucidated.

A total of 263 chemical compounds and 39 prototype compounds in the plasma of SFSY were characterized (Figure S1–S3, Tables S1–S3). To clarify the effect of SFSY treatment on Treg cell function, the function and frequency of Th, Ts, Th1, Th2, Th17, and Treg cells were detected in the well‐established rodent model of HS/R (Figure S4A) and a naïve Treg cell model. As shown in Figure 1A, SFSY treatment did not affect the transcripts of Tbx21, Gata3, and Rorc, but increased FOXP3 transcript in response to HS/R. We also found that SFSY increased the proportion of Treg cells in both peripheral blood mononuclear cells (PBMCs) and lungs (Figure 1B,C; Figures S4B and S5). Furthermore, the incubation with SFSY increased the localization of FOXP3 to the nuclei of Treg cells (Figure 1D; Figure S6). These results indicate that SFSY treatment augments FOXP3 expression, thereby promoting Treg cell function both in in vitro and in vivo.

**FIGURE 1:**
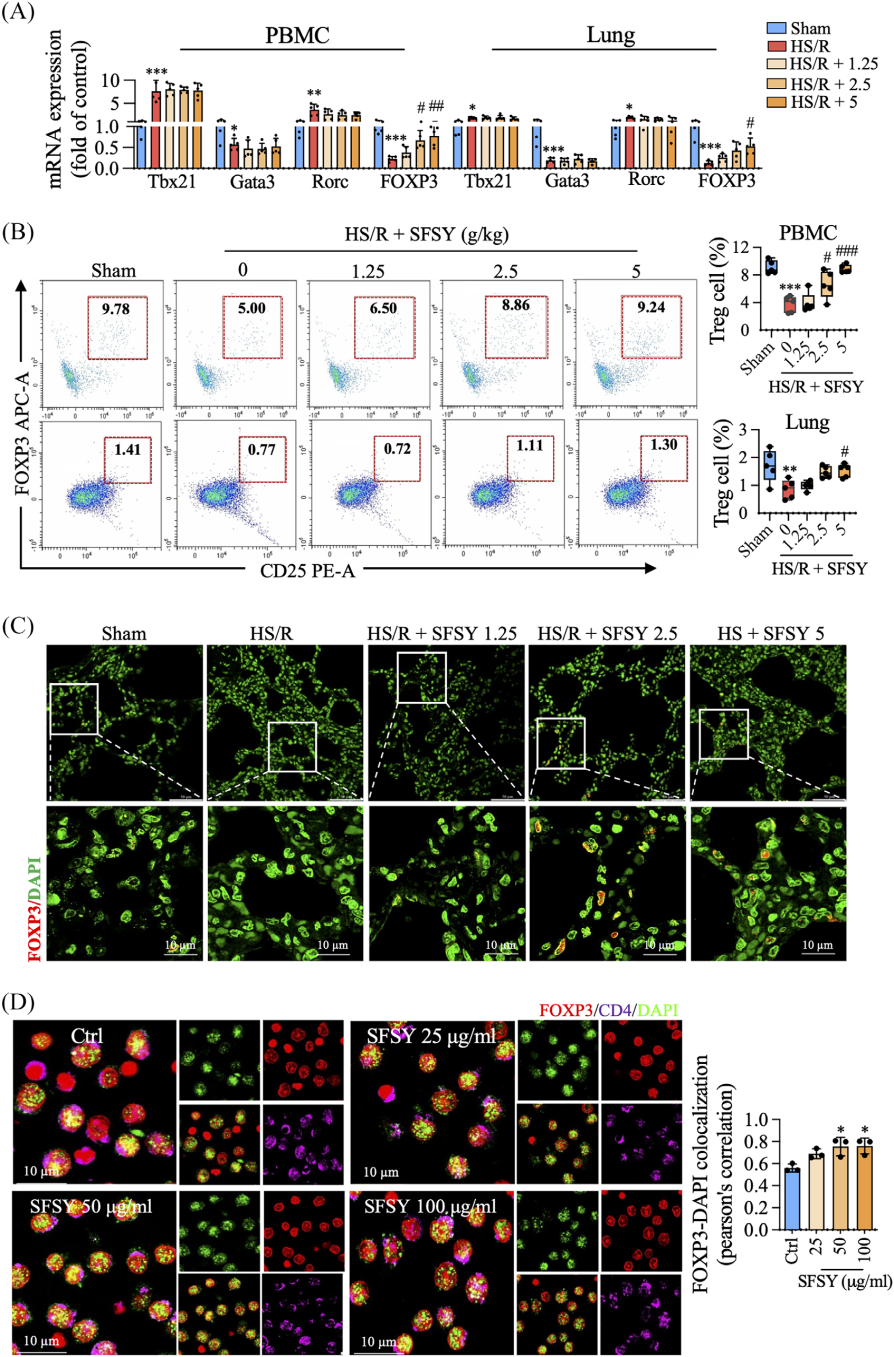
SFSY treatment improves the Treg cell function in vivo and in vitro. (A) qPCR assay was used to analyze the transcripts of Tbx21. (B) The Treg cell percentages in peripheral blood mononuclear cells (PBMCs) and lung cells were analyzed by flow cytometry. (C) The co‐localization of FOXP3 and DAPI was analyzed by immunofluorescence and confocal microscopy in the lungs. (D) The Treg cell function was analyzed by immunofluorescence staining and co‐localization analysis in Treg cells.

Additionally, SFSY treatment increased the blood pressure and heart rate (Figure 2A,B; Figure S7A). The HS/R‐induced metabolic disorders, lymphocyte depletion, and MOD (lungs, liver, kidneys, and intestine) injury were also ameliorated by SFSY treatment (Figure 2C–F; Figures S7B–S11). To explore the molecular mechanisms underlying the SFSY‐mediated Treg cell function, CD4^+^CD25^+^ Treg cells were purified from PBMCs, and transcriptomic sequencing was performed. The results of principal component and volcano map analyses showed significant differences in mRNA expression among the Sham, HS/R, and HS/R + SFSY groups (Figure 2G,H; Figure S12). SFSY significantly reduced the HS/R‐induced activation of immune‐related pathways in Treg cells (Figure 2I; Figure S13A). Further validation studies in Treg cells and lungs demonstrated that the enhancement of Treg cell function by SFSY against HS/R‐induced injury was Stat1‐, Ebi3‐, CXCL10‐, and Gbp5‐dependent manner (Figure 2J; Figures S13B–S15).

**FIGURE 2:**
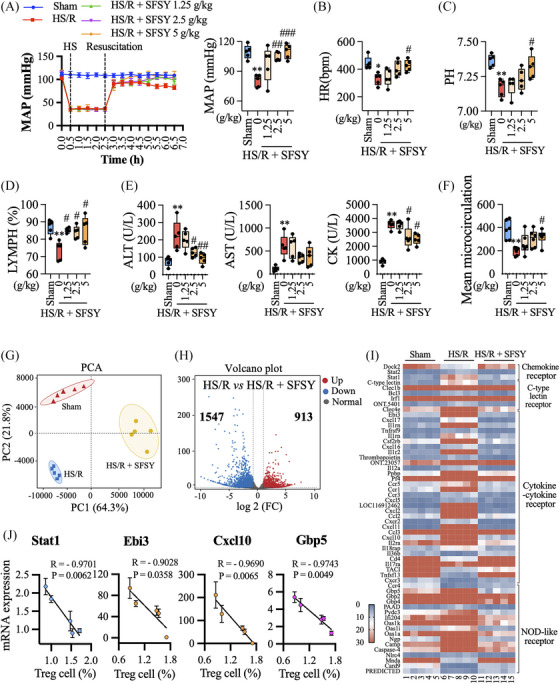
The potential molecular mechanisms underlying SFSY‐mediated Treg cell function were analyzed by transcriptomic analysis. (A, B) The MAP and heart rate were continuously monitored by a digital biological signal acquisition and processing system. (C) The blood gas and electrolytes were analyzed after the arterial blood was collected into anticoagulant tubes with heparin lithium. (D) After resuscitation, the arterial blood was collected from the aorta, and an anticoagulant with EDTA‐2K was added. The complete blood count was detected by a haematology analyzer. (E) The ALT (alanine aminotransferase), AST (aspartate aminotransferase), and CK (creatine kinase) concentrations in serum were analyzed by biochemical method. (F) The microcirculation in the intestine had been measured and analyzed by a laser speckle imaging system before the rats were sacrificed. (G) The principal component analysis showed significant differences in mRNA expression among the Sham, HS/R, and HS/R + SFSY groups. (H) The volcano plot shows the transcriptional changes identified in the HS/R group vs. the HS/R + SFSY group. (I) The heat map displays the differentially expressed genes of the immunity‐related pathways in these three groups. Red and blue represent high and low levels of expression of the indicated genes, respectively. (J) The association between differentially expressed genes and FOXP3 expression in the lungs was analyzed by correlation coefficient plots.

Next, we screened the bioactive ingredients in SFSY by integrative pharmacological screen strategy based on ingredients in plasma and phenotype experiments. Ginsenoside Ro, hypaconitine, loganic acid, secologanin, and wogonin significantly decreased the mRNA levels of IL‐6, TNF‐α, and IL‐1β in A549 cells under LPS incubation (Figure S16). Importantly, ginsenoside Ro decreased the expression of Stat1, hypaconitine reduced the levels of Stat1 and CXCL10, and loganic acid decreased the expression of Gbp5 both in A549 and Treg cells (Figure S17A). We also found that the addition of ginsenoside Ro, hypaconitine, or loganic acid increased the frequency of FOXP3^+^ cells as well as their surface expression of CD4 in Treg cells (Figure S17B,C). Stat1 can be translocated to both the nucleus and mitochondria after phosphorylation to regulate FOXP3 transcription and the mitochondria function of Treg cells.^4^, ^5^ Ginsenoside Ro or hypaconitine treatment inhibited the phosphorylation of Stat1 and promoted the expression of FOXP3 in naïve Treg cells, which were significantly blocked by fludarabine (Stat1 inhibitor; Figure 3A–C; Figure S18A). Additionally, incubation with fludarabine abolished the reduction of CXCL10 mRNA under hypaconitine incubation in Treg cells, indicating that hypaconitine inhibits the phosphorylation of Stat1 to reduce CXCL10 transcription and promote FOXP3 expression (Figure 3D). Considering that ginsenoside Ro did not affect CXCL10 transcription, we speculated that it could affect the mitochondrial function of Treg cells. We analyzed mitochondrial dynamic, mitophagy, mitochondrial biogenesis, mitochondrial apoptosis, and mitochondrial oxidative phosphorylation (OXPHOS) function, and found that ginsenoside Ro mainly augmented the mitochondrial biogenesis, balanced mitochondrial dynamic, and enhanced mitochondrial OXPHOS function in Treg cells in a Stat1‐dependent manner (Figure 3E–H; Figure S18B,C). Taken together, these results suggest that both ginsenoside Ro and hypaconitine may inhibit the phosphorylation of Stat1, but their mechanisms of enhancing Treg cell function are different.

**FIGURE 3:**
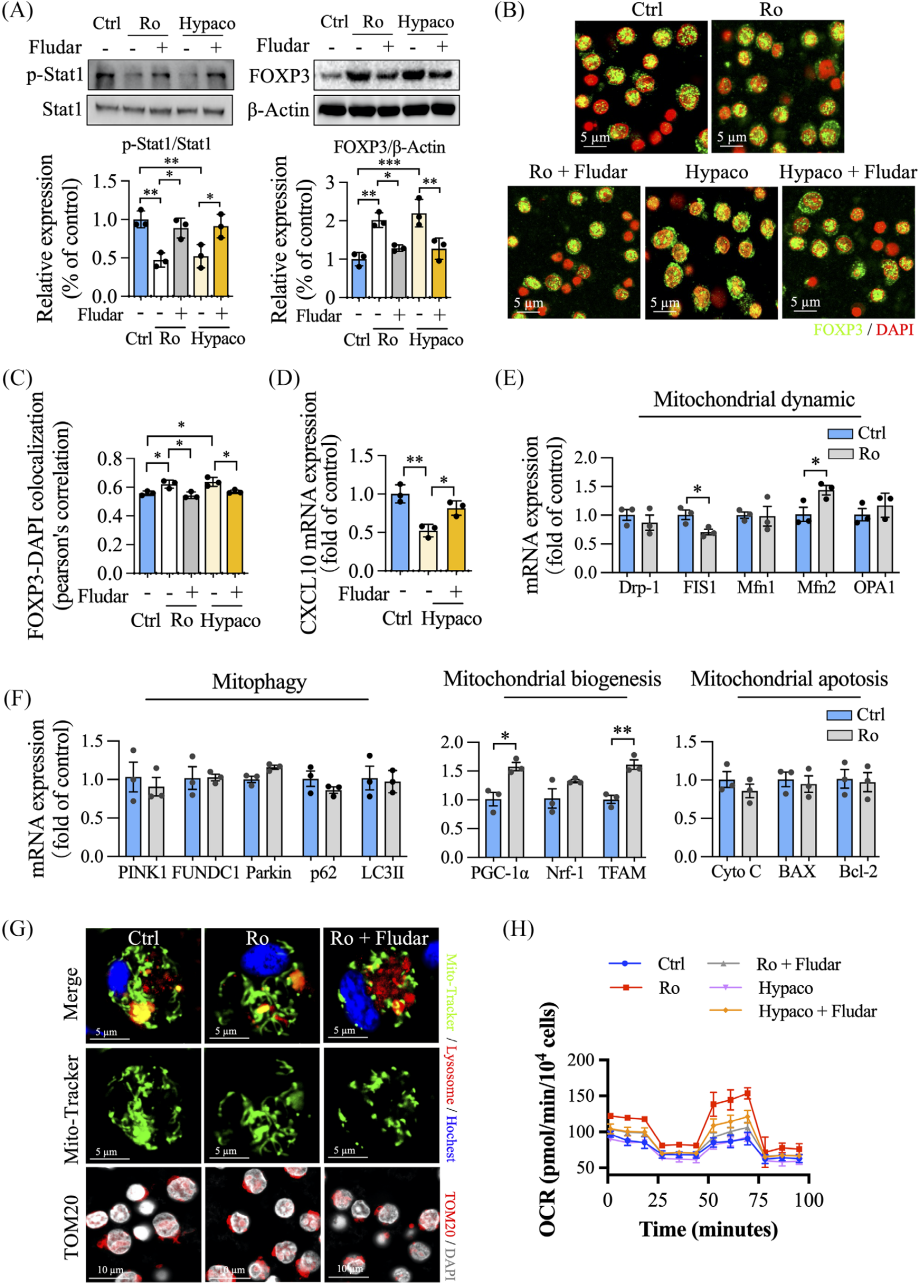
Stat1 inhibition abrogates the improved efficacy of Treg function by ginsenoside Ro or hypaconitine treatment in Treg cell polarizing conditions. (A) Immunoblot analysis of p‐Stat1, Stat1, and FOXP3 in Treg cells after ginsenoside Ro, hypaconitine, or/and fludarabine treatment under Treg cell polarizing conditions. (B, C) Immunofluorescence was used to analyze the co‐localization of FOXP3 and DAPI in Treg cells. (D) The level of CXCL10 mRNA was analyzed by qPCR assay. (E, F) The mRNA expression of mitochondrial function was analyzed by qPCR in Treg cells. (G) The Treg cells were pretreated with ginsenoside Ro or/and Fludarabine for 3 days, and the mitochondrial counts and mitochondrial fission were analyzed by living‐cell staining with MitoTracker, Lyso‐Tracker, and Hoechst 33258 probes. The expression of TOM20 was analyzed by immunofluorescence. (H) The mitochondrial function was recorded by continuous injections with inhibitors, including oligomycin, carbonyl cyanide 4‐trifluoromethoxy‐phenylhydrazone, and a mixture of antimycin A and rotenone.

Gbp5, a unique regulator of NLRP3 inflammasome activation in innate immunity, can promote GSDMD‐mediated pyroptosis.^6^ Although loganic acid had an inhibitory effect on Gbp5 transcription (Figure S17A), our western blot experiment did not show a decrease in Gbp5 expression. Therefore, we used LPS to induce a pyroptosis condition to enhance the abundance of changes in Gbp5 protein. Indeed, we found increased Gbp5 expression and cleaved‐GSDMD/GSDMD ratio in LPS‐treated Treg cells, which was abolished by treatment with loganic acid (Figure 4A). Furthermore, Gbp5 knockdown significantly ablated the effects of loganic acid treatment on decreasing pyroptosis and enhancing Treg cell function (Figure 4B–D; Figure S19A). The calcein/PI dye staining and apoptosis analysis confirmed that the circumvention of Gbp5‐mediated pyroptosis was the substantial mechanism of loganic acid treatment on Treg cell survival (Figure 4E,F; Figure S19B).

**FIGURE 4:**
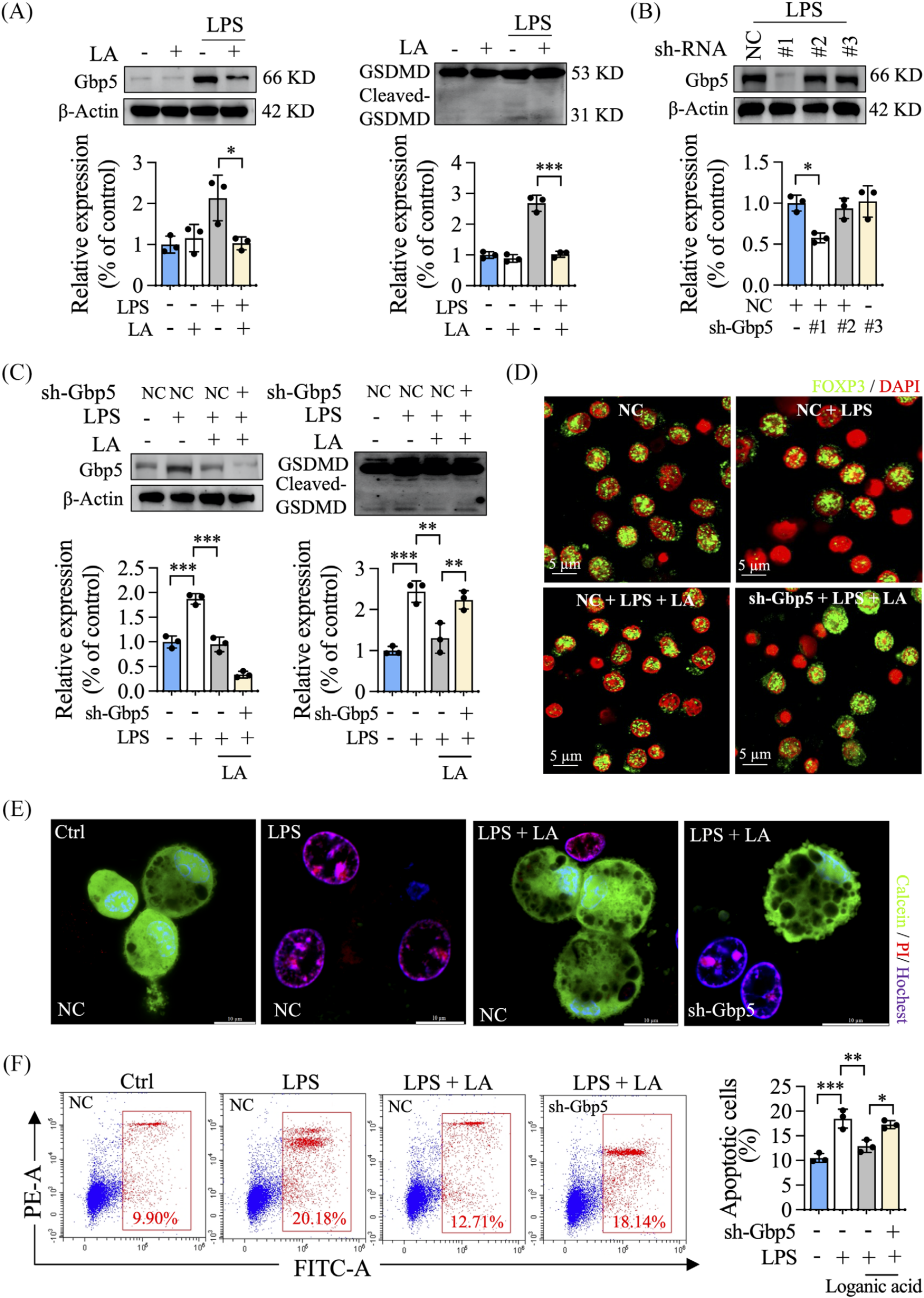
Loganic acid inhibits Gbp5‐mediated pyroptosis to enhance Treg cell function. (A) Immunoblot analysis of Gbp5, GSDMD, and FOXP3 in Treg cells after loganic acid (LA) or/and LPS incubation under Treg cell polarizing conditions. (B) Treg cells were infected with NC or sh‐Gbp5 lentivirus, and the infection efficiency was analyzed by western blot. (C) The effect of LA on decreasing Gbp5‐mediated pyroptosis was analyzed by western blot. (D) The co‐localization of FOXP3 and DAPI in Treg cells was analyzed by immunofluorescence staining. (E, F) The pyroptosis and apoptosis of the Treg cells were evaluated using calcein/propidium iodide (PI) staining and Annexin V/PI staining kits.

In conclusion, our study demonstrated that SFSY treatment promoted the stability of FOXP3, thereby enhancing Treg cell function and alleviating HS/R‐induced metabolic disorders, lymphopenia, and MOD. Mechanistically, we revealed the following: (1) ginsenoside Ro circumvented the translocation of phosphorylated Stat1 to mitochondria, thereby increasing the mitochondrial function of Treg cells; (2) hypaconitine inhibited the phosphorylation of Stat1, thereby reducing CXCL10 transcription and promoting FOXP3 expression; and (3) loganic acid mitigated the activation of Gbp5 to inhibit Treg cell pyroptosis mediated by GSDMD cleavage (Graphical abstract). Our research illustrates that bioactive compounds from SFSY enhance Treg cell function against HS/R injury via Stat1‐ and Gbp5‐dependent FOXP3 induction.

## AUTHOR CONTRIBUTIONS

Qingxia Huang: Investigation, data curation, methodology, validation, writing‐original draft, funding acquisition. Mingxia Wu: Data curation, investigation, formal analysis, writing‐original draft, visualization. Lu Ding: Investigation, formal analysis, validation, methodology. Chen Guo: Resources, data analysis, validation. Yisa Wang: data analysis, validation. Zhuo Man: Software, investigation. Hang Su: Data analysis, visualization. Jing Li: Investigation, validation. Jinjin Chen: Investigation, methodology. Yao Yao: Methodology. Zeyu Wang: Project administration. Daqing Zhao: Writing‐review & editing. Linhua Zhao: Supervision, methodology, data analysis, writing‐review & editing. Xiaolin Tong: Methodology, writing‐review & editing, supervision, funding acquisition. Xiangyan Li: Conceptualization, supervision, methodology, resources, funding acquisition, writing‐review & editing.

## CONFLICT OF INTEREST STATEMENT

The authors declare no conflict of interest.

## ETHICS STATEMENT

Animal protocols were approved by the Animal Ethics Committee of Changchun University of Chinese Medicine (Changchun, China, approval no. 2022433). All animal experiments used in this study were performed strictly in accordance with the ARRIVE guidelines 2.0 and the National Institutes of Health Guide for the Care and Use of Laboratory Animals.

## Supporting information



Supporting Information

Supporting Information

## Data Availability

The data supporting the findings of this study are available from the corresponding authors upon reasonable request.
