# Supplementary material for: Bioactive compounds from ShenFuShanYuRou decoction enhance Treg cell function against hemorrhagic shock injury via Stat1‐ and Gbp5‐dependent FOXP3 induction
Source: Clin Transl Med. 2024 Oct 11;14(10):e70047. doi: 10.1002/ctm2.70047 (PMC11469952; doi:10.1002/ctm2.70047)
Supplement: Supplementary file 1 — Supporting Information [file CTM2-14-e70047-s001.doc]

**Table S4. The information o**f the main components in SFSY identified by LC-MS/MS analysis

| **No.** | **Component name** | **Observed RT (min)** | **Formula** | **Adduct/ Charge** | **Area** | **Found Mass** | **Mass Error (ppm)** |
| --- | --- | --- | --- | --- | --- | --- | --- |
| 1 | Loganin | 5.33 | C17H26O10.HCOOH | [M-H]- | 1.01E+08 | 435.1505 | -0.7 |
| 2 | Monoside | 4.33 | C17H26O11.HCOOH | [M-H]- | 6.20E+07 | 451.1454 | -0.6 |
| 3 | D-glucopyranosyl-20(S)-protopanaxtriol | 13.63 | C37H62O10 | [M-H]- | 2.91E+07 | 665.4259 | -1.7 |
| 4 | Cornus officinalis glycoside | 7.44 | C24H30O14 | [M+NH4]+ | 1.81E+07 | 560.1972 | -0.3 |
| 5 | Secologanin or isomer | 5.2 | C17H24O10 | [M+FA-H]- | 8.00E+06 | 433.1348 | -0.9 |
| 6 | Loganin-2 | 4.26 | C17H26O10.HCOOH | [M-H]- | 4.87E+06 | 435.1502 | -1.3 |
| 7 | Perseitol Heptaacetate | 5.74 | C21H30O14 | [M+Na]+ | 4.45E+06 | 529.1525 | -0.5 |
| 8 | Dangyaoside | 5.28 | C16H22O9 | [M+H]+ | 4.06E+06 | 359.1336 | -0.2 |
| 9 | 5-hydroxymethylfurfural-1 | 2.04 | C6H6O3 | [M+H]+ | 3.48E+06 | 127.0387 | -1.8 |
| 10 | Hyperoside | 6.75 | C21H20O12 | [M-H]- | 3.02E+06 | 463.0872 | -2.1 |
| 11 | Bamboo joint ginseng saponin-2 | 12.64 | C42H66O14 | [M-H]- | 2.97E+06 | 793.4363 | -2.1 |
| 12 | Tongguanteng glycoside | 14.3 | C42H66O14 | [M-H]- | 2.71E+06 | 793.4364 | -2 |
| 13 | 4-Methylumbelliferone-2 | 4.36 | C10H8O3 | [M+H]+ | 2.68E+06 | 177.0543 | -1.9 |
| 14 | 1,7-di-O-galloyl-Dsedoheptulose | 1.58 | C14H18O11 | [M+Na]+ | 2.54E+06 | 385.0739 | -0.7 |
| 15 | 1,2,3,6-tetragalloylglucose | 6.11 | C34H28O22 | [M-H]- | 1.98E+06 | 787.0985 | -1.9 |
| 16 | D-mannose D- (+) | 0.72 | C6H12O6 | [M-H]- | 1.93E+06 | 179.0558 | -1.8 |
| 17 | Quercetin-2 | 6.97 | C15H10O7 | [M+H]+ | 1.54E+06 | 303.0496 | -1.1 |
| 18 | Melibiose | 0.71 | C12H22O11 | [M+Na]+ | 1.31E+06 | 365.1048 | -1.8 |
| 19 | Mangiferin-2 | 5.28 | C19H18O11 | [M-H]- | 1.02E+06 | 421.0768 | -1.9 |
| 20 | 1,3,6-tri-O-galloylglucose-3 | 4.58 | C27H24O18 | [M-H]- | 9.74E+05 | 635.0879 | -1.7 |
| 21 | 1,3,6-tri-O-galloylglucose-1 | 4.88 | C27H24O18 | [M-H]- | 9.33E+05 | 635.0878 | -1.8 |
| 22 | 6-Methylcoumarin-2 | 5.37 | C10H8O2 | [M+H]+ | 9.12E+05 | 161.0594 | -2.1 |
| 23 | Quercetin-3 | 8.77 | C15H10O7 | [M+H]+ | 9.08E+05 | 303.0495 | -1.6 |
| 24 | Quercetin-1 | 6.77 | C15H10O7 | [M+H]+ | 8.38E+05 | 303.0495 | -1.5 |
| 25 | Forsythia glycoside-1 | 6.6 | C29H36O15 | [M-H]- | 6.83E+05 | 623.1968 | -2.2 |
| 26 | Kaempferol-3-O-Rutinoside-2 | 6.99 | C27H30O15 | [M-H]- | 6.53E+05 | 593.1498 | -2.3 |
| 27 | Naringenin-7-O-glucoside-3 | 7.09 | C21H22O10 | [M-H]- | 6.15E+05 | 433.1129 | -2.5 |
| 28 | 4-Methylumbelliferone-1 | 5.23 | C10H8O3 | [M+H]+ | 5.85E+05 | 177.0542 | -2.2 |
| 29 | 1,3,6-tri-O-galloylglucose-2 | 5.23 | C27H24O18 | [M-H]- | 5.80E+05 | 635.0881 | -1.5 |
| 30 | Gallic acid-4-O-β-D-glucoside or isomer | 1.16 | C13H16O10 | [M-H]- | 5.42E+05 | 331.0667 | -1.2 |
| 31 | 7S-O-morroniside or isomer | 4.36 | C17H26O11 | [M+Na]+ | 5.40E+05 | 429.1364 | -0.8 |
| 32 | Korilajing-3 | 1.42 | C27H22O18 | [M-H]- | 5.37E+05 | 633.0729 | -0.7 |
| 33 | Melezitose | 0.73 | C18H32O16 | [M+Na]+ | 5.17E+05 | 527.1579 | -0.7 |
| 34 | Adenosine Monophosphate | 0.98 | C10H14N5O7P | [M+H]+ | 5.09E+05 | 348.0701 | -0.8 |
| 35 | Camphor | 7.38 | C10H16O | [M+H]+ | 4.71E+05 | 153.127 | -2.3 |
| 36 | Paeoniflorin | 5.69 | C23H28O11.HCOOH | [M-H]- | 4.29E+05 | 525.1606 | -1.5 |
| 37 | Luteolin-1 | 7.26 | C15H10O6 | [M+H]+ | 3.72E+05 | 287.0542 | -2.7 |
| 38 | Beta-penta-O-galloyl-glucose | 6.87 | C41H32O26 | [M-H]- | 3.72E+05 | 939.1093 | -1.7 |
| 39 | Zhimu saponin | 9.55 | C45H76O19.HCOOH | [M-H]- | 3.63E+05 | 965.4942 | -2.1 |
| 40 | Pineolin monomethyl ether-D-glucoside | 6.92 | C26H32O11 | [M-H]- | 3.61E+05 | 519.1859 | -2.5 |
| 41 | Naringin-4 | 7.13 | C15H12O5 | [M+H]+ | 3.56E+05 | 273.0752 | -2.1 |
| 42 | Korilajing-2 | 2.1 | C27H22O18 | [M-H]- | 3.47E+05 | 633.073 | -0.5 |
| 43 | Astragaloside | 7.26 | C21H20O11 | [M+H]+ | 3.41E+05 | 449.1071 | -1.6 |
| 44 | Rutoside | 6.69 | C27H30O16 | [M+Na]+ | 3.39E+05 | 633.1419 | -1.1 |
| 45 | 6-Methylcoumarin-1 | 4.52 | C10H8O2 | [M+H]+ | 2.53E+05 | 161.0593 | -2.7 |
| 46 | Naringenin-7-O-glucoside-2 | 6.19 | C21H22O10 | [M-H]- | 2.52E+05 | 433.1128 | -2.7 |
| 47 | Root bark glycoside | 7.56 | C21H24O10 | [M-H]- | 2.52E+05 | 435.1284 | -2.9 |
| 48 | Forsythia glycoside-2 | 6.18 | C29H36O15 | [M-H]- | 2.49E+05 | 623.1965 | -2.6 |
| 49 | Hesperidin | 7.31 | C28H34O15 | [M-H]- | 2.47E+05 | 609.1811 | -2.4 |
| 50 | Naringin-3 | 9.2 | C15H12O5 | [M+H]+ | 2.46E+05 | 273.0754 | -1.4 |
| 51 | Isorhamnetin-3-O-glucoside | 7.53 | C22H22O12 | [M-H]- | 2.45E+05 | 477.1027 | -2.3 |
| 52 | Myricetin | 7.61 | C15H10O8 | [M-H]- | 2.31E+05 | 317.0296 | -2.2 |
| 53 | Dihydrodaidzein | 8.29 | C15H10O4 | [M-H]- | 2.29E+05 | 253.0498 | -3.1 |
| 54 | Isoimperatorin | 12.64 | C16H14O4 | [M-H]- | 2.27E+05 | 269.0811 | -3 |
| 55 | 8-epigallocatechin or isomer | 5.15 | C17H26O10 | [M+NH4]+ | 2.27E+05 | 408.1859 | -1.2 |
| 56 | D-Fructose-6-phosphate disodium salt hydrate | 0.75 | C6H13O9P | [M-H]- | 2.18E+05 | 259.0218 | -2.7 |
| 57 | 5-Hydroxymethylfurfural-2 | 0.76 | C6H6O3 | [M+H]+ | 1.86E+05 | 127.0386 | -3.1 |
| 58 | Naringenin-7-O-glucoside-1 | 7.85 | C21H22O10 | [M-H]- | 1.59E+05 | 433.1126 | -3.3 |
| 59 | Harpagoside | 9.38 | C24H30O11 | [M+Na]+ | 1.53E+05 | 517.1676 | -0.9 |
| 60 | Liquiritin | 6.4 | C21H22O9 | [M-H]- | 1.51E+05 | 417.1181 | -2.4 |
| 61 | Spiraeoside-2 | 7.87 | C21H20O12 | [M-H]- | 1.50E+05 | 463.0867 | -3.3 |
| 62 | Arabinofuranosyluracil | 1 | C9H12N2O6 | [M-H]- | 1.47E+05 | 243.0618 | -2.1 |
| 63 | Luteolin-2 | 9.77 | C15H10O6 | [M+H]+ | 1.30E+05 | 287.0547 | -1.2 |
| 64 | Naringenin-2 | 6.43 | C15H12O5 | [M+H]+ | 1.26E+05 | 273.0754 | -1.2 |
| 65 | Luteolin-5 | 7.49 | C15H10O6 | [M+H]+ | 1.26E+05 | 287.0545 | -1.6 |
| 66 | Tigogenin | 11.32 | C27H44O3 | [M+H]+ | 1.22E+05 | 417.3354 | -2.1 |
| 67 | Petunidin-3-O-beta-glucopyranoside | 7.55 | C22H23O12 | [M]+ | 1.14E+05 | 479.1182 | -0.5 |
| 68 | Luteolin-4 | 7.08 | C15H10O6 | [M+H]+ | 1.12E+05 | 287.0546 | -1.4 |
| 69 | Corilagin | 3.12 | C27H22O18 | [M-H]- | 1.09E+05 | 633.0722 | -1.8 |
| 70 | Pedunculoside-2 | 11.44 | C36H58O10.HCOOH | [M-H]- | 1.07E+05 | 695.3992 | -2.9 |
| 71 | Salsoline | 1.86 | C11H15NO2 | [M+H]+ | 1.06E+05 | 194.1171 | -2.4 |
| 72 | Puerarin | 5.2 | C21H20O9 | [M+H]+ | 1.03E+05 | 417.1176 | -1.1 |
| 73 | Kaempferol 3-O-Rutinoside-1 | 7.23 | C27H30O15 | [M-H]- | 9.81E+04 | 593.1496 | -2.7 |
| 74 | Hexahydroxybiphenyl dicarbonyl-Dglucoside | 3.64 | C27H22O18 | [M-H]- | 9.79E+04 | 633.0723 | -1.6 |
| 75 | 1-Methyladenosine | 2.48 | C11H15N5O4 | [M+H]+ | 9.45E+04 | 282.119 | -2.5 |
| 76 | Eriodictyol-2 | 8.14 | C15H12O6 | [M-H]- | 9.28E+04 | 287.055 | -3.8 |
| 77 | Plantamajoside | 5.79 | C29H36O16 | [M-H]- | 8.21E+04 | 639.1928 | -0.4 |
| 78 | Naringenin-1 | 7.89 | C15H12O5 | [M+H]+ | 7.93E+04 | 273.0757 | -0.1 |
| 79 | Eriodictyol-1 | 7.3 | C15H12O6 | [M-H]- | 7.39E+04 | 287.0552 | -3.1 |
| 80 | Pedunculoside-1 | 10.45 | C36H58O10.HCOOH | [M-H]- | 7.36E+04 | 695.3985 | -4 |
| 81 | Wogonin | 8.77 | C16H12O5 | [M+H]+ | 7.32E+04 | 285.0754 | -1.1 |
| 82 | Esculin | 3.92 | C15H16O9 | [M-H]- | 7.11E+04 | 339.0711 | -3.2 |
| 83 | Hesperetin | 7.33 | C16H14O6 | [M+H]+ | 6.84E+04 | 303.0861 | -0.8 |
| 84 | Formononetin | 10.46 | C16H12O4 | [M-H]- | 5.86E+04 | 267.0655 | -2.8 |
| 85 | Isoliquiritigenin | 8.22 | C15H12O4 | [M+H]+ | 5.73E+04 | 257.0805 | -1.2 |
| 86 | Luteolin-3 | 6.45 | C15H10O6 | [M+H]+ | 5.61E+04 | 287.0545 | -2 |
| 87 | Syringetin-3-O-galactoside | 7.55 | C23H24O13 | [M-H]- | 5.52E+04 | 507.1128 | -3.1 |
| 88 | Liquiritigenin | 8.19 | C15H12O4 | [M-H]- | 5.48E+04 | 255.0651 | -4.6 |
| 89 | Pedunculoside-3 | 10.86 | C36H58O10.HCOOH | [M-H]- | 4.92E+04 | 695.3989 | -3.3 |
| 90 | Nobiletin | 11.92 | C21H22O8 | [M+H]+ | 4.89E+04 | 403.138 | -1.9 |
| 91 | Apigenin | 9.73 | C15H10O5 | [M-H]- | 3.80E+04 | 269.0446 | -3.4 |
| 92 | Isorhamnetin | 9.95 | C16H12O7 | [M-H]- | 3.78E+04 | 315.0505 | -1.8 |
| 93 | Scutellarin | 7.55 | C21H18O12 | [M+H]+ | 3.39E+04 | 463.087 | -0.3 |
| 94 | Mangiferin-1 | 7.07 | C19H18O11 | [M-H]- | 3.30E+04 | 421.0762 | -3.3 |
| 95 | L (+)-Arginine | 0.71 | C6H14N4O2 | [M+H]+ | 1.56E+07 | 175.1188 | -0.9 |
| 96 | Citric Acid | 0.93 | C6H8O7 | [M-H]- | 1.46E+07 | 191.0199 | 0.8 |
| 97 | Quercetin 3-O-glucuronide | 6.93 | C21H18O13 | [M-H]- | 1.45E+07 | 477.0669 | -1.2 |
| 98 | Quininic acid-2 | 0.7 | C7H12O6 | [M-H]- | 1.38E+07 | 191.056 | -0.5 |
| 99 | L-Malic acid | 0.78 | C4H6O5 | [M-H]- | 1.21E+07 | 133.0143 | 0.8 |
| 100 | loganic acid | 4.16 | C16H24O10 | [M-H]- | 8.90E+06 | 375.1295 | -0.5 |
| 101 | p-Coumalic acid-3 | 5.18 | C9H8O3 | [M-H]- | 6.73E+06 | 163.0399 | -1 |
| 102 | L-Pyroglutamic acid | 0.99 | C5H7NO3 | [M+H]+ | 5.37E+06 | 130.0496 | -1.8 |
| 103 | Maleic acid | 0.78 | C4H4O4 | [M-H]- | 4.68E+06 | 115.0037 | 0.3 |
| 104 | Gallic acid | 1.45 | C7H6O5 | [M-H]- | 3.37E+06 | 169.0144 | 0.6 |
| 105 | Caffeic acid-3 | 4.48 | C9H8O4 | [M-H]- | 2.82E+06 | 179.0349 | -0.5 |
| 106 | Ellagic acid | 6.73 | C14H6O8 | [M-H]- | 2.70E+06 | 300.9984 | -2 |
| 107 | Caffeic acid-1 | 4.72 | C9H8O4 | [M-H]- | 2.35E+06 | 179.0348 | -0.9 |
| 108 | Asiatic acid | 13.41 | C30H48O5 | [M-H]- | 1.97E+06 | 487.3414 | -3 |
| 109 | Tyrosine | 1.02 | C9H11NO3 | [M+H]+ | 1.91E+06 | 182.0809 | -1.6 |
| 110 | 2-3-ethenyl-5-methoxycarbonyl-2 | 5.84 | C17H24O11 | [M-H]- | 1.58E+06 | 403.1243 | -0.7 |
| 111 | 2-Hydroxycinnamic acid | 1.02 | C9H8O3 | [M+H]+ | 1.18E+06 | 165.0543 | -2 |
| 112 | Phenylalanine | 1.76 | C9H11NO2 | [M+H]+ | 1.16E+06 | 166.086 | -1.4 |
| 113 | Leucine | 1.12 | C6H13NO2 | [M+H]+ | 1.16E+06 | 132.1016 | -2.5 |
| 114 | Caffeic acid-2 | 3.77 | C9H8O4 | [M-H]- | 1.13E+06 | 179.0348 | -1.3 |
| 115 | p-Coumalic acid-4 | 5.83 | C9H8O3 | [M-H]- | 1.11E+06 | 163.0399 | -0.8 |
| 116 | Quercetin-3-O-β-D-glucuronic acid or isomer | 6.48 | C21H18O13 | [M-H]- | 1.04E+06 | 477.0667 | -1.5 |
| 117 | Syringic Acid | 5.48 | C9H10O5 | [M-H]- | 9.82E+05 | 197.0453 | -1.1 |
| 118 | Piscidic Acid | 2.57 | C11H12O7 | [M-H]- | 7.90E+05 | 255.0508 | -1.1 |
| 119 | Proline | 0.72 | C5H9NO2 | [M+H]+ | 7.82E+05 | 116.07 | -4.8 |
| 120 | Coniferic acid-2 | 5.6 | C10H10O4 | [M-H]- | 7.59E+05 | 193.0503 | -1.6 |
| 121 | L-Tyrosine | 4.75 | C9H11NO3 | [M+H]+ | 6.83E+05 | 182.0808 | -1.9 |
| 122 | 3-O-Feruloylquinic acid | 5.7 | C17H20O9 | [M-H]- | 6.78E+05 | 367.1026 | -2.4 |
| 123 | 4-Coumaric acid-1 | 5.88 | C9H8O3 | [M+H]+ | 6.60E+05 | 165.0544 | -1.3 |
| 124 | (Z)-9,12,13-trihydroxyoctadec-15-enoic acid | 11.16 | C18H34O5 | [M+Na]+ | 6.59E+05 | 353.23 | 0.4 |
| 125 | Quininic acid-1 | 5.35 | C7H12O6 | [M-H]- | 6.35E+05 | 191.0559 | -1.3 |
| 126 | Pipecolic acid | 0.77 | C6H11NO2 | [M+H]+ | 4.86E+05 | 130.0862 | -0.5 |
| 127 | Sodium pantothenate-2 | 2.55 | C9H17NO5 | [M+H]+ | 4.58E+05 | 220.1176 | -1.5 |
| 128 | Fat-soluble vitamin | 0.94 | C6H5NO2 | [M+H]+ | 4.32E+05 | 124.0388 | -4.1 |
| 129 | Chlorogenic acid-1 | 4.48 | C16H18O9 | [M-H]- | 4.28E+05 | 353.0871 | -1.9 |
| 130 | p-Coumalic acid-1 | 4.39 | C9H8O3 | [M-H]- | 4.23E+05 | 163.0397 | -2.1 |
| 131 | Coniferic acid-1 | 4.33 | C10H10O4 | [M-H]- | 4.14E+05 | 193.0501 | -2.6 |
| 132 | Azelaic acid | 7.52 | C9H16O4 | [M-H]- | 3.43E+05 | 187.0971 | -2.5 |
| 133 | Mucic acid | 0.73 | C6H10O8 | [M-H]- | 3.39E+05 | 209.0298 | -2.2 |
| 134 | P-Coumalic acid-2 | 3.83 | C9H8O3 | [M-H]- | 2.86E+05 | 163.0398 | -1.5 |
| 135 | Isoferulic acid | 6.29 | C10H10O4 | [M-H]- | 2.65E+05 | 193.0502 | -2.4 |
| 136 | 3-Indoleacetic acid | 2.41 | C10H9NO2 | [M+H]+ | 2.23E+05 | 176.0704 | -1.4 |
| 137 | Tryptophan | 2.94 | C11H12N2O2 | [M-H]- | 2.14E+05 | 203.0822 | -1.8 |
| 138 | Protocatechuic acid-3 | 13.18 | C18H18O2 | [M-H]- | 2.13E+05 | 265.1228 | -2.3 |
| 139 | Catechol | 2.74 | C6H6O2 | [M-H]- | 1.65E+05 | 109.0293 | -1.8 |
| 140 | Sodium pantothenate-1 | 3.05 | C9H17NO5 | [M+H]+ | 1.63E+05 | 220.1177 | -1.1 |
| 141 | Protocatechuic acid-1 | 2.74 | C7H6O4 | [M-H]- | 1.63E+05 | 153.0192 | -1.1 |
| 142 | Sinapic acid | 5.88 | C11H12O5 | [M+H]+ | 1.54E+05 | 225.0753 | -2.1 |
| 143 | Sodium 4-hydroxy-benzoate | 6.75 | C7H6O3 | [M+H]+ | 1.50E+05 | 139.0385 | -3.3 |
| 144 | A-hexahydrocyclopenta pyran-4-carboxylic acid | 6.37 | C16H24O9 | [M-H]- | 1.46E+05 | 359.1339 | -2.5 |
| 145 | 3-Phenyllactic acid | 5.71 | C9H10O3 | [M-H]- | 1.42E+05 | 165.0554 | -1.7 |
| 146 | Salicylic acid-2 | 6.9 | C7H6O3 | [M-H]- | 1.27E+05 | 137.0241 | -2.4 |
| 147 | 4-Coumaric acid-2 | 1.05 | C9H8O3 | [M+H]+ | 1.21E+05 | 165.0545 | -1 |
| 148 | Abscisic acid | 8.34 | C15H20O4 | [M-H]- | 1.15E+05 | 263.128 | -3.2 |
| 149 | Protocatechuic acid-2 | 3.61 | C7H6O3 | [M-H]- | 1.15E+05 | 137.0242 | -1.7 |
| 150 | Oxybenzoic acid | 8.35 | C23H30O12 | [M-H]- | 1.14E+05 | 497.1653 | -2.3 |
| 151 | Chlorogenic acid-2 | 5.07 | C16H18O9 | [M-H]- | 1.08E+05 | 353.0871 | -2 |
| 152 | SINAPIC ACID | 4.74 | C11H12O5 | [M-H]- | 1.07E+05 | 223.0605 | -2.9 |
| 153 | Succinic Acid | 1.12 | C4H6O4 | [M-H]- | 1.04E+05 | 117.0189 | -3.8 |
| 154 | 9-(2,3-dihydroxypropoxy)-9-oxononanoic acid | 7.12 | C12H22O6 | [M-H]- | 1.03E+05 | 261.1338 | -2.3 |
| 155 | Oxan-2-yl oxycyclopentyl acetic acid | 8.06 | C18H30O8 | [M-H]- | 8.96E+04 | 373.1855 | -3.3 |
| 156 | Oxyphenyl prop-2-enoic acid | 6.24 | C16H20O9 | [M-H]- | 8.81E+04 | 355.1022 | -3.4 |
| 157 | N-acetyltryptophan | 6.32 | C13H14N2O3 | [M-H]- | 5.74E+04 | 245.0924 | -3.1 |
| 158 | Salicylic acid-1 | 1.93 | C7H6O3 | [M-H]- | 3.88E+04 | 137.0241 | -2.6 |
| 159 | Ursolic Acid-1 | 17.43 | C30H48O3 | [M+H]+ | 2.38E+04 | 457.3673 | -0.6 |
| 160 | Ginsenoside Rh1-4 | 11.51 | C36H62O9.HCOOH | [M-H]- | 4.82E+07 | 683.436 | -2.3 |
| 161 | Ginsenoside Ro | 11.92 | C48H76O19 | [M-H]- | 3.03E+07 | 955.4885 | -2.4 |
| 162 | Ginsenoside Rh1-3 | 10.38 | C36H62O9.HCOOH | [M-H]- | 1.91E+07 | 683.436 | -2.3 |
| 163 | Ginsenoside Rg2-3 | 11.32 | C42H72O13 | [M-H]- | 1.61E+07 | 783.4875 | -3.3 |
| 164 | 20(R)-ginsenoside Rg3-2 | 11.2 | C42H72O13 | [M+FA-H]- | 1.38E+07 | 829.4932 | -2.8 |
| 165 | Pseudoginsenoside F11-1 | 10.63 | C42H72O14 | [M-H]- | 1.15E+07 | 799.4829 | -2.5 |
| 166 | 20(R)-ginsenoside Rg3-2 | 14.95 | C42H72O13 | [M-H]- | 1.05E+07 | 783.4881 | -2.5 |
| 167 | Adenosine | 1.27 | C10H13N5O4 | [M+H]+ | 9.79E+06 | 268.104 | -0.2 |
| 168 | Ginsenoside F4 or isomer | 13.38 | C42H70O12 | [M+FA-H]- | 9.58E+06 | 811.4832 | -2.1 |
| 169 | Pseudoginsenoside F11-2 | 10.76 | C42H72O14 | [M-H]- | 7.94E+06 | 799.4831 | -2.3 |
| 170 | Ginsenoside Rg2-2 | 10.23 | C42H72O13.HCOOH | [M-H]- | 7.87E+06 | 829.4935 | -2.4 |
| 171 | Ginsenoside Rg2-7 | 14.95 | C42H72O13.HCOOH | [M-H]- | 7.43E+06 | 829.4934 | -2.5 |
| 172 | 20(R)-ginsenoside Rf2 or isomer-2 | 8.59 | C42H74O14 | [M+FA-H]- | 7.16E+06 | 847.5045 | -1.9 |
| 173 | Ginsenoside F2 | 15.04 | C42H72O13.HCOOH | [M-H]- | 6.87E+06 | 829.4931 | -2.9 |
| 174 | 20(R)-ginsenoside Rf2 or isomer-1 | 8.42 | C42H74O14 | [M+FA-H]- | 6.48E+06 | 847.504 | -2.4 |
| 175 | 20(R)-ginsenoside Rg3-1 | 13.74 | C42H72O13 | [M-H]- | 4.51E+06 | 783.4882 | -2.3 |
| 176 | Ginsenoside Rg2-4 | 10.23 | C42H72O13 | [M-H]- | 4.45E+06 | 783.4882 | -2.3 |
| 177 | 20(R)-notoginsenoside R2-2 | 10.897 | C41H70O13 | [M+FA-H]- | 4.33E+06 | 815.478 | -2.3 |
| 178 | Ginsenoside F5 | 11.17 | C41H70O13 | [M-H]- | 4.22E+06 | 769.4726 | -2.3 |
| 179 | Pseudoginsenoside F11-0 | 9.67 | C42H72O14 | [M-H]- | 3.42E+06 | 799.4833 | -2.1 |
| 180 | Ginsenoside Rg5 or isomer | 12.92 | C42H70O13 | [M+FA-H]- | 3.33E+06 | 827.4779 | -2.4 |
| 181 | Ginsenoside Rg1-1 | 9.68 | C42H72O14.HCOOH | [M-H]- | 3.23E+06 | 845.4887 | -2 |
| 182 | Ginsenoside Rh1-1 | 12.22 | C36H62O9.HCOOH | [M-H]- | 2.55E+06 | 683.4358 | -2.6 |
| 183 | 20(R)-notoginsenoside R2-1 | 10.1 | C41H70O13 | [M+FA-H]- | 2.20E+06 | 815.4782 | -2 |
| 184 | Ginsenoside Rg2-1 | 13.74 | C42H72O13.HCOOH | [M-H]- | 1.74E+06 | 829.493 | -3 |
| 185 | Ginsenoside Rh1-2 | 12.65 | C36H62O9.HCOOH | [M-H]- | 1.67E+06 | 683.436 | -2.4 |
| 186 | Panaxydol | 12.79 | C17H24O2 | [M+H]+ | 1.60E+06 | 261.1845 | -1.7 |
| 187 | Pseudoginsenoside F11-3 | 11.42 | C42H72O14 | [M-H]- | 1.02E+06 | 799.4826 | -3 |
| 188 | Ginsenoside Rg2-6 | 12.04 | C42H72O13.HCOOH | [M-H]- | 9.70E+05 | 829.4931 | -2.9 |
| 189 | Guanosine | 1.29 | C10H13N5O5 | [M+H]+ | 8.08E+05 | 284.0989 | -0.3 |
| 190 | Ginsenoside Rg2-5 | 12.05 | C42H72O13 | [M-H]- | 7.33E+05 | 783.4877 | -2.9 |
| 191 | Ginsenoside Rg1-2 | 11.45 | C42H72O14.HCOOH | [M-H]- | 6.81E+05 | 845.4878 | -3.1 |
| 192 | Ginsenoside Rh1 | 11.51 | C36H62O9 | [M-H]- | 5.99E+05 | 637.4299 | -3.4 |
| 193 | Pseudoginsenoside RT5 | 10.72 | C36H62O10.HCOOH | [M-H]- | 4.58E+05 | 699.4305 | -2.8 |
| 194 | Pseudoginsenoside F11-5 | 12.95 | C42H72O14 | [M-H]- | 3.64E+05 | 799.4826 | -2.9 |
| 195 | Albiflorin | 5.36 | C23H28O11 | [M+H]+ | 3.37E+05 | 481.1695 | -1.9 |
| 196 | Pseudoginsenoside F11-4 | 11.85 | C42H72O14 | [M-H]- | 2.93E+05 | 799.4821 | -3.6 |
| 197 | Pseudoginsenoside-3 | 10.75 | C36H62O10 | [M+H]+ | 2.49E+05 | 655.4406 | -1.5 |
| 198 | Panaxadiol-2 | 13.77 | C30H52O3 | [M+H]+ | 2.09E+05 | 461.3984 | -1.2 |
| 199 | Panaxadiol-1 | 12.37 | C30H52O3 | [M+H]+ | 1.77E+05 | 461.3983 | -1.4 |
| 200 | Pseudoginsenoside-2 | 9.79 | C36H62O10 | [M+H]+ | 1.53E+05 | 655.4408 | -1.1 |
| 201 | Notoginsenoside Ft1 | 14.53 | C47H80O17H.COOH | [M-H]- | 1.48E+05 | 961.5349 | -3 |
| 202 | Pedunculoside | 10.89 | C36H58O10.NH3 | [M+H]+ | 1.48E+05 | 668.4362 | -0.9 |
| 203 | Rhoifolin | 4.97 | C27H30O14 | [M+H]+ | 1.36E+05 | 579.1703 | -0.9 |
| 204 | Amygdalin | 6.09 | C20H27NO11.NH3 | [M+H]+ | 1.26E+05 | 475.1932 | 2.1 |
| 205 | Ginsenoside-2 | 14.64 | C48H82O18.HCOOH | [M-H]- | 1.12E+05 | 991.5456 | -2.7 |
| 206 | Nodakenin | 6.97 | C20H24O9 | [M+H]+ | 9.05E+04 | 409.1487 | -1.4 |
| 207 | Ginsenoside-3 | 11.09 | C48H82O18.HCOOH | [M-H]- | 8.73E+04 | 991.546 | -2.3 |
| 208 | Benzoylpaeoniflorin | 9.61 | C30H32O12.NH3 | [M+H]+ | 7.71E+04 | 602.2224 | -1.3 |
| 209 | Pseudoginsenoside-1 | 7.4 | C36H62O10 | [M+H]+ | 7.10E+04 | 655.442 | 0.7 |
| 210 | Araloside A | 10.83 | C42H66O14 | [M-H]- | 5.08E+04 | 793.4354 | -3.2 |
| 211 | Ginsenoside-1 | 14.03 | C48H82O18.HCOOH | [M-H]- | 5.00E+04 | 991.545 | -3.3 |
| 212 | Choline | 0.71 | C5H14NO | [M]+ | 3.44E+06 | 104.1066 | -3.4 |
| 213 | Methyl 3,4,5-trihydroxybenzoate | 4.57 | C20H20O14 | [M-H]- | 1.85E+06 | 483.0771 | -1.9 |
| 214 | DIPALNDKWHCLEA-UHFFFAOYSA-N-1 | 7.37 | C16H28O7 | [M+Na]+ | 1.53E+06 | 355.1722 | -1.4 |
| 215 | DVWKMCPPEMUHBE-RAGYRXETSA-N | 7.08 | C26H34O11 | [M+NH4]+ | 1.34E+06 | 540.2435 | -0.8 |
| 216 | Adenine-2 | 1.27 | C5H5N5 | [M+H]+ | 1.01E+06 | 136.0616 | -1.5 |
| 217 | AHYOMNWKYGMYMB-CIEFDVMPSA-N | 6.37 | C26H34O11 | [M-H]- | 9.64E+05 | 521.2017 | -2.2 |
| 218 | BFCZPWYLRHFBNO-DTJAAUDXSA-N | 1.31 | C12H20O8 | [M+Na]+ | 9.46E+05 | 315.1047 | -1.2 |
| 219 | LBRPLJCNRZUXLS-YTMAOMSOSA-N | 10.33 | C26H30N2O8 | [M+H]+ | 9.25E+05 | 499.2073 | -0.4 |
| 220 | PNYQFRZBMVRYFC-CGWYSGAGSA-N | 6.79 | C22H20O13 | [M-H]- | 9.14E+05 | 491.0819 | -2.5 |
| 221 | Adenine-1 | 0.8 | C5H5N5 | [M+H]+ | 6.64E+05 | 136.0616 | -1.4 |
| 222 | Glutathione (oxidized form) | 0.92 | C20H32N6O12S2 | [M-H]- | 6.37E+05 | 611.1441 | -0.9 |
| 223 | YNMFDPCLPIMRFD-PEXUZNNCSA-N | 6.23 | C26H28O16 | [M-H]- | 5.67E+05 | 595.1292 | -2.2 |
| 224 | LEEYYHJQGXOXHR-MYZNJVEUSA-N-1 | 10.842 | C54H86O24 | [M-H]- | 5.55E+05 | 1117.5407 | -2.6 |
| 225 | RFFYIBOJHUSIGD-PHDUQKSESA-N | 8.89 | C21H36O10 | [M+NH4]+ | 5.40E+05 | 466.264 | -1.4 |
| 226 | QZMAEZWZCGBZFK-VPQYALDZSA-N-1 | 11.75 | C48H76O19 | [M-H]- | 5.19E+05 | 955.4883 | -2.6 |
| 227 | DIPALNDKWHCLEA-UHFFFAOYSA-N-2 | 6.64 | C16H28O7 | [M+Na]+ | 4.59E+05 | 355.1723 | -1.3 |
| 228 | 6-(hydroxymethyl) oxan-2-yl oxypropan | 4.89 | C15H20O8 | [M-H]- | 4.06E+05 | 327.1078 | -2.3 |
| 229 | KBDOXLBZVFQTMH-RXRQAHPVSA-N | 8.93 | C24H30O12 | [M+Na]+ | 3.22E+05 | 533.1624 | -1 |
| 230 | LEEYYHJQGXOXHR-MYZNJVEUSA-N-2 | 11.63 | C54H86O24 | [M-H]- | 2.91E+05 | 1117.5401 | -3.1 |
| 231 | 2-hydroxyquinoline | 2.36 | C9H7NO | [M+H]+ | 2.84E+05 | 146.0597 | -2.4 |
| 232 | Oxyoxan-2-yl methyl 3,4,5-trihydroxybenzoate | 3.3 | C20H20O14 | [M-H]- | 2.59E+05 | 483.0772 | -1.6 |
| 233 | Riboflavin | 5.37 | C17H20N4O6 | [M+H]+ | 2.40E+05 | 377.1451 | -1.3 |
| 234 | 6-(hydroxymethyl) oxan-2-yl oxypropan | 4.11 | C15H20O8 | [M-H]- | 1.90E+05 | 327.1079 | -2 |
| 235 | 6-(hydroxymethyl) oxan-3-yl trihydroxybenzoate | 7.02 | C28H24O16 | [M-H]- | 1.74E+05 | 615.0976 | -2.6 |
| 236 | OVMSOCFBDVBLFW-ZZMVMVDNSA-N | 8.67 | C31H38O11 | [M+Na]+ | 1.68E+05 | 609.2298 | -1.4 |
| 237 | RWEVZSYWIIZZFV-UHFFFAOYSA-N | 7.58 | C26H34O11 | [M+Na]+ | 1.33E+05 | 545.1983 | -2 |
| 238 | 5-ethenyl-3-hydroxy-4-(3-hydroxyprop-1-en-2-yl) | 6.09 | C23H32O9 | [M+Na]+ | 1.25E+05 | 475.1933 | -1.2 |
| 239 | LIVNOUBJFOXZOR-YMILTQATSA-N | 4.57 | C16H22O9 | [M+NH4]+ | 1.04E+05 | 376.16 | -0.5 |
| 240 | IFIQVSCCFRXSJV-ZIZFEDMCSA-N | 10.31 | C30H48O6 | [M-H]- | 1.04E+05 | 503.3361 | -3.4 |
| 241 | IUCHKMAZAWJNBJ-DKAZIEIMSA-N | 15.318 | C36H56O9 | [M-H]- | 5.02E+04 | 631.3831 | -3.2 |
| 242 | YVMUBJPWZKUGBC-NUTNSJPXSA-N | 9.91 | C22H30O11 | [M+Na]+ | 4.63E+04 | 493.1695 | 3 |
| 243 | IFIQVSCCFRXSJV-ZIZFEDMCSA-N | 11.4 | C30H48O6 | [M-H]- | 4.42E+04 | 503.3356 | -4.4 |
| 244 | PUETUDUXMCLALY-UHFFFAOYSA-N-2 | 6.17 | C20H26O6 | [M-H]- | 4.30E+04 | 361.1642 | -4 |
| 245 | 3,4,5-trihydroxy-6-(hydroxymethyl) oxan-2-yl] | 8.19 | C36H58O12 | [M+Na]+ | 4.22E+04 | 705.3814 | -1 |
| 246 | PUETUDUXMCLALY-UHFFFAOYSA-N-1 | 7.65 | C20H26O6 | [M-H]- | 3.92E+04 | 361.1643 | -3.7 |
| 247 | Benzoylmesaconine | 7.23 | C31H43NO10 | [M+H]+ | 2.00E+08 | 590.2956 | -0.6 |
| 248 | Fuziline | 4.59 | C24H39NO7 | [M+H]+ | 6.20E+07 | 454.28 | 0.1 |
| 249 | Benzoylaconitine | 7.62 | C32H45NO10 | [M+H]+ | 3.53E+07 | 604.3112 | -0.8 |
| 250 | Benzoylhypaconine | 7.89 | C31H43NO9 | [M+H]+ | 3.41E+07 | 574.3009 | -0.2 |
| 251 | Hypaconitine | 9.12 | C33H45NO10 | [M+H]+ | 2.72E+07 | 616.3113 | -0.4 |
| 252 | Bullatine B or isomer | 4.79 | C24H39NO6 | [M+H]+ | 1.96E+07 | 438.2849 | -0.3 |
| 253 | Mesaconine or isomer | 3.56 | C24H39NO9 | [M+H]+ | 1.70E+07 | 486.2697 | 0 |
| 254 | Benzoylmesaconine or isomer | 6.37 | C31H43NO11 | [M+H]+ | 1.57E+07 | 606.2907 | -0.3 |
| 255 | Songorine or isomer | 3.88 | C22H31NO3 | [M+H]+ | 9.97E+06 | 358.2375 | -0.6 |
| 256 | Mesaconitine | 8.71 | C33H45NO11 | [M+H]+ | 6.03E+06 | 632.306 | -0.9 |
| 257 | Chasmanine | 5.7 | C25H41NO6 | [M+H]+ | 2.84E+06 | 452.3003 | -0.9 |
| 258 | Sn-Glycero-3-phosphocholine | 0.73 | C8H21NO6P | [M]+ | 1.33E+06 | 258.1094 | -2.5 |
| 259 | Senbusine C or isomer | 4.4 | C24H39NO7 | [M+FA-H]- | 1.28E+06 | 498.2698 | -2 |
| 260 | Tetrahydroberberine | 7.49 | C20H17NO4 | [M+H]+ | 1.18E+06 | 336.1226 | -1.4 |
| 261 | Sinapoylcholine | 4.53 | C16H24NO5 | [M]+ | 2.51E+05 | 310.1644 | -1.7 |
| 262 | Indole-3-aldehyde | 2.97 | C9H7NO | [M+H]+ | 1.80E+05 | 146.0598 | -1.8 |
| 263 | 10-OH benzoylaconine or isomer | 6.76 | C32H45NO11 | [M+H]+ | 1.72E+05 | 620.3057 | -1.3 |

Table S5. The information of the main components in plasma identified by LC-MS/MS analysis

| **NO.** | **Component name** | **Observed RT (min)** | **Formula** | **Adduct/Charge** | **Area** | **Found Mass** | **Mass Error (ppm)** |
| --- | --- | --- | --- | --- | --- | --- | --- |
| 1 | D-mannose D-(+) | 0.67 | C6H12O6 | [M-H]- | 7.21E+04 | 179.0556 | -2.7 |
| 2 | Melibiose | 0.68 | C12H22O11 | [M+Na]+ | 2.14E+05 | 365.1053 | -0.4 |
| 3 | Adenosine Monophosphate | 0.72 | C10H14N5O7P | [M+H]+ | 3.04E+04 | 348.0706 | 0.6 |
| 4 | Melezitose | 0.73 | C18H32O16 | [M+Na]+ | 2.80E+04 | 527.1582 | -0.1 |
| 5 | D-Fructose-6-phosphate disodium salt hydrate | 0.75 | C6H13O9P | [M-H]- | 7.08E+04 | 259.0212 | -4.6 |
| 6 | Morroniside | 4.31 | C17H26O11.HCOOH | [M-H]- | 1.36E+05 | 451.1452 | -1.1 |
| 7 | Secologanin or isomer | 5.2 | C17H24O10 | [M+FA-H]- | 7.83E+03 | 433.1342 | -2.1 |
| 8 | Loganetin | 5.32 | C17H26O10.HCOOH | [M-H]- | 2.02E+05 | 435.1501 | -1.5 |
| 9 | Cornuside | 7.38 | C24H30O14 | [M-H]- | 6.32E+04 | 541.1557 | -1.1 |
| 10 | Dihydrodaidzein | 8.29 | C15H10O4 | [M-H]- | 1.91E+04 | 253.05 | -2.5 |
| 11 | Wogonin | 8.55 | C16H12O5 | [M+H]+ | 6.52E+03 | 285.0751 | -2.3 |
| 12 | Apigenin | 9.41 | C15H10O5 | [M-H]- | 1.48E+04 | 269.0449 | -2.4 |
| 13 | Choline | 0.67 | C5H14NO | [M]+ | 2.98E+05 | 104.1065 | -4.3 |
| 14 | Glutathione (oxidized form) | 0.73 | C20H32N6O12S2 | [M-H]- | 2.46E+05 | 611.1434 | -2.1 |
| 15 | Riboflavin | 5.33 | C17H20N4O6 | [M+H]+ | 1.58E+04 | 377.1455 | -0.1 |
| 16 | Sn-Glycero-3-phosphocholine | 0.71 | C8H21NO6P | [M]+ | 8.07E+04 | 258.1098 | -1 |
| 17 | Indole-3-aldehyde | 2.92 | C9H7NO | [M+H]+ | 1.64E+05 | 146.0598 | -1.8 |
| 18 | Fuziline | 4.52 | C24H39NO7 | [M+H]+ | 9.40E+03 | 454.28 | 0.1 |
| 19 | Bullatine B or isomer | 4.77 | C24H39NO6 | [M+H]+ | 2.23E+03 | 438.2839 | -2.6 |
| 20 | Benzoylmesaconine | 7.17 | C31H43NO10 | [M+H]+ | 3.14E+04 | 590.296 | 0 |
| 21 | Benzoylaconitine | 7.54 | C32H45NO10 | [M+H]+ | 2.37E+03 | 604.3119 | 0.4 |
| 22 | Benzoylhypaconine | 7.82 | C31H43NO9 | [M+H]+ | 2.14E+03 | 574.3007 | -0.7 |
| 23 | Hypaconitine | 9.09 | C33H45NO10 | [M+H]+ | 4.31E+03 | 616.3114 | -0.4 |
| 24 | Pipecolic acid | 0.59 | C6H11NO2 | [M+H]+ | 5.18E+04 | 130.0857 | -4.4 |
| 25 | Proline | 0.7 | C5H9NO2 | [M+H]+ | 1.41E+05 | 116.0701 | -4.3 |
| 26 | Quinic acid-2 | 0.71 | C7H12O6 | [M-H]- | 1.45E+05 | 191.0554 | -3.7 |
| 27 | L-Malic acid | 0.74 | C4H6O5 | [M-H]- | 3.89E+04 | 133.014 | -1.7 |
| 28 | 2-Hydroxycinnamic acid | 0.84 | C9H8O3 | [M+H]+ | 1.73E+05 | 165.0545 | -0.9 |
| 29 | Tyrosine | 0.84 | C9H11NO3 | [M+H]+ | 2.30E+05 | 182.081 | -1.1 |
| 30 | Citric Acid | 0.85 | C6H8O7 | [M-H]- | 1.76E+06 | 191.0197 | -0.2 |
| 31 | Leucine | 0.87 | C6H13NO2 | [M+H]+ | 5.27E+05 | 132.1016 | -2.4 |
| 32 | Phenylalanine | 1.68 | C9H11NO2 | [M+H]+ | 6.52E+05 | 166.0861 | -1 |
| 33 | Sodium pantothenate-2 | 2.47 | C9H17NO5 | [M+H]+ | 1.86E+05 | 220.1178 | -0.8 |
| 34 | Tryptophan | 2.91 | C11H12N2O2 | [M-H]- | 4.51E+05 | 203.0824 | -0.9 |
| 35 | loganic acid | 4.09 | C16H24O10 | [M-H]- | 1.78E+04 | 375.1291 | -1.5 |
| 36 | Ellagic acid | 6.74 | C14H6O8 | [M-H]- | 2.35E+04 | 300.9986 | -1.4 |
| 37 | Salicylic acid | 6.94 | C7H6O3 | [M-H]- | 2.76E+04 | 137.0243 | -1 |
| 38 | 9-(2,3-dihydroxypropoxy)-9-oxononanoic acid | 7.16 | C12H22O6 | [M-H]- | 5.54E+03 | 261.1338 | -2.2 |
| 39 | Ginsenoside Ro | 11.91 | C48H76O19 | [M-H]- | 4.52E+03 | 955.4887 | -2.2 |

Table S6. The absolute quantitative results of the protoype compounds of SFSY in plasma

| **Component Name** | **FragmentMass** | **RetentionTime** | **DP** | **CE** | **Area**  **(QC)** | **Area**  **(Sham)** | **Area**  **(HS/R)** | **Area(HS/R+**  **SFSY-1)** | **Area(HS/R+SFSY-2)** |
| --- | --- | --- | --- | --- | --- | --- | --- | --- | --- |
| Ginsenoside Ro | 955.491 | 11.91 | -80 | -20 | 6.49E+04 | 2.16E+04 | 1.62E+04 | 6.22E+04 | 6.06E+04 |
| Phenylalanine | 120.087 | 1.68 | 80 | 40 | 3.38E+07 | 1.98E+07 | 3.20E+07 | 3.17E+07 | 3.49E+07 |
| Proline | 70.069 | 0.7 | 80 | 40 | 8.21E+06 | 5.85E+06 | 8.54E+06 | 8.02E+06 | 8.14E+06 |
| Tryptophan | 116.052 | 2.91 | -80 | -40 | 4.92E+06 | 6.28E+06 | 3.98E+06 | 3.91E+06 | 4.03E+06 |
| Citric acid | 87.012 | 0.85 | -80 | -40 | 4.48E+06 | 2.79E+06 | 4.50E+06 | 4.87E+06 | 4.81E+06 |
| 2-Hydroxycinnamic acid | 95.049 | 0.84 | 80 | 40 | 4.22E+06 | 3.68E+06 | 4.03E+06 | 3.99E+06 | 3.95E+06 |
| Quininic acid-2 | 111.009 | 0.71 | -80 | -40 | 2.84E+06 | 1.62E+06 | 2.70E+06 | 2.88E+06 | 2.88E+06 |
| Salicylic acid-2 | 93.035 | 6.94 | -80 | -40 | 1.96E+06 | 1.16E+06 | 1.96E+06 | 1.99E+06 | 2.01E+06 |
| Leucine | 86.103 | 0.87 | 80 | 40 | 1.34E+06 | 7.08E+05 | 1.35E+06 | 1.18E+06 | 1.24E+06 |
| Tyrosine | 123.047 | 0.84 | 80 | 40 | 1.10E+06 | 9.13E+05 | 9.74E+05 | 9.70E+05 | 1.03E+06 |
| Hexahydropyridine carboxylic acid | 69.982 | 0.59 | 80 | 40 | 4.53E+05 | 3.00E+05 | 5.03E+05 | 4.46E+05 | 4.70E+05 |
| Sodium pantothenate-2 | 90.055 | 2.47 | 80 | 40 | 2.44E+05 | 1.42E+05 | 2.63E+05 | 2.62E+05 | 2.63E+05 |
| Loganic acid | 69.035 | 4.09 | -80 | -40 | 4.66E+04 | N/A | N/A | 8.74E+04 | 8.97E+04 |
| 9-oxononanoic acid | 125.098 | 7.16 | -80 | -40 | 3.50E+04 | 3.49E+04 | 3.26E+04 | 3.81E+04 | 3.83E+04 |
| Tannic acid | 283.997 | 6.74 | -80 | -45 | 2.61E+04 | 4.83E+04 | 4.64E+04 | 3.31E+04 | 2.77E+04 |
| L-malic acid | 78.961 | 0.74 | -80 | -40 | 1.76E+04 | 1.26E+04 | 1.83E+04 | 2.07E+04 | 2.17E+04 |
| Indole-3-aldehyde | 91.053 | 2.92 | 80 | 40 | 5.84E+07 | 7.25E+07 | 4.46E+07 | 4.42E+07 | 4.64E+07 |
| sn-Glycero-3-phosphocholine | 141.07 | 0.71 | 80 | 40 | 1.07E+06 | 6.62E+05 | 1.07E+06 | 1.16E+06 | 1.17E+06 |
| Benzoyl neoaconitine | 540.261 | 7.17 | 80 | 45 | 3.11E+05 | 2.40E+04 | 2.66E+04 | 4.27E+05 | 4.36E+05 |
| Bullatine B or isomer | 438.305 | 4.77 | 80 | 40 | 2.59E+05 | N/A | N/A | 3.99E+05 | 4.10E+05 |
| Fuziling | 436.271 | 4.52 | 80 | 40 | 2.18E+05 | 1.62E+04 | 1.66E+04 | 2.88E+05 | 3.15E+05 |
| Hypaconitine | 556.279 | 9.09 | 80 | 40 | 1.09E+05 | 4.57E+03 | 3.92E+03 | 1.92E+05 | 2.05E+05 |
| Benzoyl diaconitine | 542.276 | 7.82 | 80 | 45 | 9.57E+04 | 1.35E+04 | 1.23E+04 | 9.90E+04 | 9.83E+04 |
| Benzoyl aconitine | 554.274 | 7.54 | 80 | 50 | 3.90E+04 | 5.46E+03 | 5.41E+03 | 4.07E+04 | 4.34E+04 |
| Choline | 58.065 | 0.67 | 80 | 40 | 2.19E+07 | 1.83E+07 | 2.11E+07 | 2.59E+07 | 2.36E+07 |
| Riboflavin | 243.089 | 5.33 | 80 | 40 | 1.05E+06 | 4.97E+05 | 1.15E+06 | 1.15E+06 | 1.19E+06 |
| Glutathione (oxidized form) | 306.076 | 0.73 | -80 | -40 | 9.85E+05 | 3.62E+05 | 4.95E+05 | 1.51E+06 | 1.70E+06 |
| Melezitose | 527.158 | 0.69 | 80 | 40 | 9.21E+05 | 6.48E+04 | 8.13E+05 | 7.74E+05 | 7.68E+05 |
| Adenosine Monophosphate | 136.061 | 0.72 | 80 | 40 | 5.48E+05 | 2.42E+06 | 1.11E+05 | 4.15E+05 | 3.60E+05 |
| D-Fructose-6-phosphate | 78.959 | 0.75 | -80 | -40 | 4.58E+05 | 1.79E+05 | 3.67E+05 | 5.97E+05 | 6.33E+05 |
| Daidzein | 253.051 | 8.29 | -80 | -40 | 2.34E+05 | 1.22E+05 | 2.51E+05 | 2.24E+05 | 2.37E+05 |
| Wogonin | 270.053 | 8.55 | 80 | 40 | 2.09E+05 | 6.68E+04 | 2.39E+05 | 2.22E+05 | 2.39E+05 |
| Apigenin | 133.03 | 9.41 | -80 | -40 | 1.76E+05 | 8.25E+04 | 1.86E+05 | 1.81E+05 | 1.94E+05 |
| Melibiose | 191.04 | 0.68 | 80 | 40 | 1.73E+05 | 1.80E+05 | 1.19E+05 | 1.65E+05 | 1.65E+05 |
| D-mannose D-(+) | 59.014 | 0.67 | -80 | -40 | 1.61E+05 | 9.80E+04 | 1.30E+05 | 1.70E+05 | 1.72E+05 |
| Morroniside | 155 | 4.31 | -80 | -50 | 1.50E+05 | N/A | N/A | 2.71E+05 | 2.52E+05 |
| Cornuside | 169.019 | 7.38 | -80 | -45 | 1.31E+05 | 2.26E+05 | 1.60E+05 | 1.34E+05 | 1.11E+05 |
| Strychnoside | 101.025 | 5.32 | -80 | -40 | 5.86E+04 | N/A | N/A | 1.11E+05 | 1.09E+05 |
| Secologanin | 297.004 | 5.2 | -80 | -40 | 2.88E+04 | N/A | 3.01E+04 | 3.06E+04 | 3.14E+04 |
