# Supplementary material for: Bioactive compounds from ShenFuShanYuRou decoction enhance Treg cell function against hemorrhagic shock injury via Stat1‐ and Gbp5‐dependent FOXP3 induction
Source: Clin Transl Med. 2024 Oct 11;14(10):e70047. doi: 10.1002/ctm2.70047 (PMC11469952; doi:10.1002/ctm2.70047)
Supplement: Supplementary file 2 — Supporting Information [file CTM2-14-e70047-s002.doc]

**Supporting Information for**

Bioactive compounds from ShenFuShanYuRou decoction enhance Treg cell function against hemorrhagic shock injury via Stat1- and Gbp5-dependent FOXP3 induction

Qingxia Huang^1,2^, Mingxia Wu^2^, Lu Ding^2,1^, Chen Guo^2^, Yisa Wang^2^, Zhuo Man^3^, Hang Su^2^, Jing Li^2^, Jinjin Chen^2^, Yao Yao^2^, Zeyu Wang^2^, Daqing Zhao^2^, Linhua Zhao^4*^, Xiaolin Tong^4*^, Xiangyan Li^2*^

^1^ Research Center of Traditional Chinese Medicine, College of Traditional Chinese Medicine, Changchun University of Chinese Medicine, Changchun, Jilin, 130021, China

^2^ Northeast Asia Research Institute of Traditional Chinese Medicine, Key Laboratory of Active Substances and Biological Mechanisms of Ginseng Efficacy, Ministry of Education, Jilin Provincial Key Laboratory of Bio-Macromolecules of Chinese Medicine, Changchun University of Chinese Medicine, Changchun, Jilin, 130117, China

^3^ SCIEX China, Beijing 100015, China

^4^ Northeast Asia Research Institute of Traditional Chinese Medicine, Changchun University of Chinese Medicine, Changchun, Jilin, 130117, China

*Corresponding authors: Linhua Zhao, Xiaolin Tong, Xiangyan Li, Northeast Asia Research Institute of Traditional Chinese Medicine, Changchun University of Chinese Medicine, Changchun, Jilin, 130117, China. Tel.: +86-431-86763803, Fax: +86-431-86763803. Email: melonzhao@163.com; tongxiaolin@vip.163.com; xiangyan_li1981@163.com.

**Table of Contents**

[1. Supplementary Materials and Methods 3](#__RefHeading___Toc166823314)

[*1.1 Chemicals and reagents* 3](#__RefHeading___Toc166823315)

[*1.2 Mass spectrometric analysis* 3](#__RefHeading___Toc166823316)

[*1.3 Animal experiments and approval* 5](#__RefHeading___Toc166823317)

[*1.4 Experimental HS/R model and treatment* 5](#__RefHeading___Toc166823318)

[*1.5 Hematological analysis* 6](#__RefHeading___Toc166823319)

[*1.6 Microcirculatory imaging* 6](#__RefHeading___Toc166823320)

[*1.7 T cell sorting and Treg cell culture* 7](#__RefHeading___Toc166823321)

[*1.8 Flow cytometry analysis* 7](#__RefHeading___Toc166823322)

[*1.9 Transcriptome analysis of Treg cells* 8](#__RefHeading___Toc166823323)

[*1.10 A549 cell culture* *and drug incubation* 9](#__RefHeading___Toc166823324)

[*1.11 Hematoxylin and eosin staining* 9](#__RefHeading___Toc166823325)

[*1.12* *Quantitative real‐time PCR* 9](#__RefHeading___Toc166823326)

[*1.13 Spearman correlation analysis* 10](#__RefHeading___Toc166823327)

[*1.14 Immunofluorescence staining* 10](#__RefHeading___Toc166823328)

[*1.15 Western blot analysis* 11](#__RefHeading___Toc166823329)

[*1.16 Lentivirus infection* 11](#__RefHeading___Toc166823330)

[*1.17 Mitochondrial respiration analysis* 11](#__RefHeading___Toc166823331)

[*1.18 Mito-Tracker and Calcein/PI staining* 12](#__RefHeading___Toc166823332)

[*1.19* *Quantification and statistical analysis* 12](#__RefHeading___Toc166823333)

[*1.20 Data availability* 13](#__RefHeading___Toc166823334)

[2 Supplementary Figures 14](#__RefHeading___Toc166823335)

[3. Supplementary Tables 34](#__RefHeading___Toc166823336)

[4. Supplementary Reference 51](#__RefHeading___Toc166823337)

# 1. Supplementary Materials and Methods

# *1.1 Chemicals and reagents*

The crude herbal drugs in SFSY decoction (*Panax ginseng C. A. Mey, Aconitum carmichaelii Debx*. and *Cornus officinalis Sieb. et Zucc.*) were purchased from Tongrentang (Beijing, China). The chemical composition standards 20(S)-ginsenoside Rg2 (52286-74-5), ginsenoside Ro (34367-04-9), 20(S)-ginsenoside Rh1 (63223-86-9), 20(R)-ginsenoside Rg3 (38243-03-7), ginsenoside Rg3 (14197-60-5), benzoylhypaconitine (63238-66-4), benzoylaconitine (466-24-0), benzoylmesaconitine (63238-67-5), fuziline (80665-72-1), gallic acid (149-91-7), morroniside (25406-64-8), loganin (18524-94-2), cornuside (131189-57-6), oleanolic acid (508-02-1), ursolic acid (77-52-1), 5-hydroxymethylfurfural (67-47-0) were purchased from the Chengdu Desite Biotechnology (Chengdu, Sichuang, China). Fludarabine (NSC 118218) was purchased from MedChemExpress (MCE, Shanghai, China). Antibodies against p-Stat1 (phospho S727, ab109461), FOXP3 (ab215206), Gbp5 (ab313390), and GSDMD (ab219800) were purchased from Abcam (Cambridge, MA, USA). Antibodies against Stat1 (#9172), TOM20 (#42406) and β-Actin (#3700) were obtained from Cell Signaling Technology (Beverly, MA, USA). Antibodies against CD4 (MA1-81588) and FOXP3 (PA1-46126) were purchased from Thermo Fisher Scientific (Waltham, MA, USA). Flow cytometry antibodies against FITC anti-CD3 (201403), PerCP/Cyanine5.5 anti-CD4 (201520), PE anti-CD8 (200608), PE anti-CD25 (202105), Alexa Fluor® 647 anti-FOXP3 (320014) were purchased from Biolegend (San Diego, CA, USA).

# *1.2 Mass spectrometric analysis*

According to the dosage of SFSY in *the Sixth Edition Diagnosis and Treatment Program of Novel Coronavirus Pneumonia* issued by the State Administration of Traditional Chinese Medicine, three crude herbal drugs in SFSY (*Panax ginseng C. A. Mey* 15 g, *Aconitum carmichaelii Debx.* 10 g and *Cornus officinalis Sieb. et Zucc.* 15 g) were immersed in water for 30 min, and decocted twice with 10-fold amount water for 50 min. After filtration, two decoctions were combined, evaporated, and lyophilized. Ten batches of extracts were prepared using the same method, and fingerprint analysis by the high-performance liquid chromatograph showed a repeatability of 90% between each batch (Fig. S1A).

The prototype compounds of SFSY in the lyophilized extract and plasma after intragastric treatment with SFSY were characterized using the ExionLC-ZenoTOF^TM^ 7600 liquid chromatography-quadrupole tandem time-of-flight mass spectrometry system (LC-Q-TOF-MS, SCIEX, Framingham, MA, USA). The quantification of prototype compounds in the plasma was performed using the Triple Quad™ 6500 system (SCIEX). The lyophilized extract was extracted with 10% methanol in an ultrasonic bath for 40 min. The protein in plasma samples were precipitated with methanol and acetonitrile in a 1:1 ratio. The obtained solutions were centrifuged and filtered through a 0.22 μm membrane filter prior to injection.

Exion LC chromatographic conditions: Phenomenex C18 chromatographic column (2.1 × 100 mm) with 0.05% formic acid aqueous solution (A) and CH3OH: CAN (1:1) (B) as mobile phases. The flow rate was 0.3 mL/min. The injection volume of the sample was 2 µL. Maximum pressurization of the system > 18000psi. ZenoTOF Mass spectrometry conditions: the ESI collection method was used, and data was collected in both positive and negative ion scanning modes. MS Level 1 Quality Range: 80-1500 m/z. MS/MS Level 2 Fragment Collection Mode: 30 MS/MS, 50-1500 m/z. The dynamic background subtraction mode was activated to obtain more efficient secondary sub ions. Secondary collision energy: 40 ± 20 eV (collecting three different collision energy spectra to obtain overall and abundant information on sub ion fragments). The temperature of the ion source was 550 ℃. The TCM library, X500R&OS software (SCIEX) and OS&MS Dial software were used to process and correct MS data.

# *1.3 Animal experiments and approval*

Male Sprague Dawley rats (200-220 g) at eight weeks of age, were purchased from the Animal Core Facility of Changchun Yisi Experimental Animal Technology Co. Ltd (Changchun, Jilin, China). Animal protocols were approved by the Animal Ethics Committee of Changchun University of Chinese Medicine (Changchun, China, approval No. 2022433). All animal experiments used in this study were performed strictly in accordance with the ARRIVE guidelines 2.0 and National Institutes of Health Guide for the Care and Use of Laboratory Animals. The rats were maintained on a 12h light/dark cycle at 23-25°C with access to sterile pellet diet and water ad libitum.

# *1.4 Experimental HS/R model and treatment*

All rats used in this study were randomly assigned to groups followed by pretreatment with SFSY and model establishment with at least 10 animals per group. The rats in the Sham and HS/R groups received sterile water, and the rats in the HS /R + SFSY group were intragastrically administered twice with SFSY (1.25-5 g/kg/day, dissolved in sterile saline) prior to HS and resuscitation, respectively.

The HS/R model was generated and utilized as previously described. Briefly, the rats were sedated with isoflurane inhalation (1.5%-2.5%) by an animal anesthesia ventilator system (RWD Life science, Shenzhen, Guangzhou, China) and anesthetized with pentobarbital (40 mg/kg, i.p.). Body temperature was maintained at 37°C on a heated operating pad. The wounds were bathed with 1% lidocaine throughout the surgical procedure to reduce postoperative pain. All efforts were taken to minimize the number of animals used and minimize pain, distress or discomfort. After the induction of anesthesia, disinfection and analgesia, the right and left femoral arteries of the rats were cannulated with PE polyethylene tubing containing 2 IU/mL of heparin. The left femoral artery cannula was connected to a BL-420 digital biological signal acquisition and processing system (TECHMAN, Chengdu, Sichuan, China) for continuous monitoring of the mean arterial pressure (MAP) and heart rate. After cannulation, rats were observed until stabilization of the MAP at 100 ± 10 mmHg. Blood was withdrawn from the right femoral artery and collected until the MAP reached 30-40 mmHg, which was maintained for 2 h or 4 h. The collected blood was kept at 4 °C. The resuscitation was performed with the collected blood and Ringer’s Lactate equal to two volumes of shed blood. In the sham group, an identical surgical procedure was performed but without withdrawal of blood and fluid resuscitation. The rats were sacrificed prior to collected tissues by intraperitoneal injection of sodium pentobarbital (180 mg/kg).

# *1.5 Hematological analysis*

After resuscitation, the arterial blood was collected from aorta and added anticoagulant with EDTA-2K for blood count analysis, and added heparin lithium for blood gas and electrolyte analysis. The complete blood count was detected by a hematology analyzer (ProCyte Dx, IDEXX Laboratories, Westbrook, ME, USA). The levels of blood gas and electrolyte were performed by a VetStat ™ analyzer (IDEXX Laboratories). After the whole blood analyses were completed, the serum centrifuged from blood was subjected to biochemical analysis for AST, ALT, LDH and CK level (BS-240VET, Mindray Animal Technology, Shenzhen, Guangzhou, China).

# *1.6 Microcirculatory imaging*

After resuscitation for 4 h, the lower abdomen of the rats was incised and the entire intestine was expanded. The microcirculation in the mesentery was recorded and analyzed by a RFLSI ZW laser speckle imaging system (RWD Life science). To avoid possible bias, the analysis of the mean microcirculation was blinded.

# *1.7 T cell sorting and Treg cell culture*

Rat PBMCs were isolated from buffy coats by Ficoll density gradient centrifugation at 500g. Spleens were collected from rat and grinded into a single cell suspension. Naïve CD4^+^CD25^+^ T cells were enriched from the PBMCs and splenocytes using the EasySep^TM^ rat CD4^+^ T cell isolation kit (#19642, STEMCELL technologies, Vancouver, Canada) and EasySep^TM^ release mouse PE positive selection Kit (#17656, STEMCELL), according to manufacturer’s recommendations. The PE-CD25 antibody in this PE positive selection Kit has been replaced with the rat antibody (202105, Biolegend). The purity was assessed by flow cytometry analysis and CD4^+^CD25^+^ cells were typically > 90%. Purified naïve CD4^+^CD25^+^ T cells (0.6 × 10^6^ cells/mL) were cultured in RPMI 1640 medium containing 10% fetal bovine serum (FBS; CLARK Bioscience, Claymont, DE, USA), 100 U/ml penicillin and 100 µg/ml streptomycin (Biosharp, Hefei, Anhui, China) and 50 μM β-mercaptoethanol on plates pre-bounded with anti-CD3 (4 μg/mL, 201401, BioLegend) and anti-CD28 (2 μg/mL, 200902, BioLegend) for 3-6 days, as indicated in each experiment. During expansion, fresh culture medium was added every 3 days. IL-2 (20 ng/mL, HY-P70718, MCE) and TGF-β (5 ng/mL, HY-P7117, MCE) were added to the culture medium for Treg cell differentiation. The differentiation efficiency of naïve Treg cells was evaluated by the percentage of FOXP3. For drug treatment experiments, vehicle control (DMSO), therapeutic ingredients or inhibitor were added to cultures on the 3rd day.

# *1.8 Flow cytometry analysis*

The PBMCs and lungs were processed to form a single-cell suspension and then placed through a 40-μm filter to remove impurities. The gating strategies were provided in Fig. S5-Fig. S6. For surface marker staining, cells were treated with red blood cell lysate, and then washed, resuspended in staining buffer consisting of 2% FBS and incubated with specific antibodies for 30 min at 4 °C in darkness, including FITC anti-CD3 (1:200), PerCP/Cyanine5.5 anti-CD4 (1:200), PE anti- CD8 Antibody (1:200), and PE anti-CD25 (1:100). For FOXP3 staining, cells were incubated with fixation-permeabilization buffer, washed with permeabilization buffer (424401, Biolegend), and then stained with Alexa Fluor® 647 anti-FOXP3 antibody (1:100). For apoptosis staining, the Annexin V-FITC probe was added to the cells for 15 min and PI probe was added for 5 min at room temperature in the dark. The apoptotic cell percentage was defined as the sum of percentages for early and late apoptotic cells. The frequency of Th, Ts, Treg, and apoptotic cells were analyzed by flow cytometry using a CytoFLEX (Beckman Coulter, Shanghai, China).

# *1.9 Transcriptome analysis of Treg cells*

Transcriptional profiling of the CD4^+^CD25^+^ Treg cells, isolated from PBMCs after HS/R operation or SFSY treatment, was performed using the Oxford Nanopore Technologies (ONT). Briefly, total RNA was isolated from Treg cells using Trizol reagent and was prepared for cDNA libraries. Totle RNA (1 µg) samples with OD 260/280 ratios of 1.8-2.0 were used for transcriptome analyses. 1 µg total RNA was reversed transcriptase enrich and then subjected to ONT adaptor ligation using T4 DNA ligase. Agencourt XP beads was used for DNA purification and the final cDNA libraries were added to FLO-MIN109 flow cells system. The raw reads on PromethION platform were first filtered with minimum average read quality score = 7 and minimum read length = 500 bp. After mapping to rRNA database prior to discarding Ribosomal RNA, full-length, non-chimiric transcripts were determined by searching for the primer at both ends of reads. The full-length reads were mapped to the reference transcriptome sequence after removing the redundant and finding the fusion transcript. Each group has five biological replicates. The differential mRNA expression analysis of two groups was performed using the DESeq2 R package (1.6.3). Genes were considered differentially expressed if the FDR < 0.01 and fold change ≥ 2. To control the false discovery rate, the *P* values were adjusted using the Benjamini and Hochberg’s approach, and an adjusted *P* < 0.01 was used as a cutoff to define the significance. Hierarchical clustering was utilized to present the selected significant differentially expressed genes. Gene Ontology (GO) enrichment analyses were performed to characterize the functionally-related genes using the GOseq R packages based Wallenius non-central hyper-geometric distribution. KEGG analyses were performed to understand the molecular-level information in Treg cells and the KOBAS software was used to test the statistical enrichment of differential expression genes. All raw RNA-seq data are available in the NCBI’s Gene Expression Omnibus database (GEO series accession number: GSE236472).

# *1.10 A549 cell culture* *and drug incubation*

A549 (human lung cancer cell line) cells were obtained from the cell bank of the American Type Culture Collection (ATCC, Manassas, VA, USA), cultured in high-glucose DMEM supplemented with 10% FBS, 100 U/ml penicillin, and 100 mg/ml streptomycin at 37 °C in a humidified incubator under 5% CO_2_. When the confluence was about 80% at passage 4-6, the A549 cells were pretreated with different ingredients in plasma of SFSY at 2.5-10 µM for 48 h, and/or 20 µg/ml LPS incubation for 24 h to induce an *in vitro* inflammatory model.

## *1.11 Hematoxylin and eosin staining*

After embedding in paraffin blocks, 5-μm-thick paraffin sections were stained with hematoxylin and eosin (H&E) to examine the histopathological changes by a pathologist blinded to the experiment, according to the operating manual. The slides were dehydrated, mounted, and acquired by a digital scanning microscope imaging system (M8 Digital Microscope &Slide Scanner, Precipoint, Thuringia, Germany).

## *1.12* *Quantitative real‐time PCR*

Total RNA was extracted using Trizol reagent method (Thermo Fisher Scientific), and RNA concentration and purity were assessed using ultramicro spectrophotometer (NanoDrop2000, Thermo Fisher). The cDNA synthesis was carried out by reverse transcription with equal amounts of RNA using a commercial cDNA synthesis kit (TIANGEN BIOTECH, Beijing, China). Afterwards, quantitative PCR was performed on a CFX96 Real-Time PCR system (Hercules, CA, USA) using QuantiTect SYBR Green PCR reagent (TIANGEN) following manufacturer’s instructions. The relative gene expression levels were calculated using the threshold cycle and the 2^−∆∆CT^ method. β-Actin was set as a reference. All primer sequences used are shown in Table S4- S6.

## *1.13 Spearman correlation analysis*

The correlation between mRNA expression and Treg cell frequency were analyzed by the spearman correlation coefficient. Correlations with *P* < 0.05 and *R* > 0.09 were considered significant and the line chart were used to display the correlations determined.

## *1.14 Immunofluorescence staining*

Treg cells were grown on glass coverslips and fixed in 4% paraformaldehyde for 30 min at room temperature and then permeabilized with 0.2% Triton X-100 in PBST for no more than 10 min at 4 °C. Then, the cells or lung sections after antigen repair were blocked with 4% BSA for 1 h and then incubated with antibodies against CD4 (1:200), FOXP3 (1:300), and TOM20 (1:100) overnight at 4 °C. After washing with PBST, the samples were stained with Alexa Fluor 488-labeled anti-rabbit (A0423, Beyotime Institute of Biotechnology, Shanghai, China, 1:200) and Cy3-labeled anti-mouse (A0521, Beyotime, 1:300) secondary antibodies for 1 h at room temperature in the dark. Finally, the nuclei were counterstained with DAPI stain for 10 min (Beyotime). The procedure also included negative controls with omission of the primary antibody and these controls did not show any immunoreaction. The images were captured using a Leica STELLARIS 5 confocal laser scanning microscope system (Leica, Wetzlar, Germany).

## *1.15* *Western blot analysis*

The Treg cells were lysed with RIPA buffer and centrifuged at 12,000 rpm for 15 min at 4 °C to remove the cell debris. The protein samples were separated by 10% or 12% PAGE-SDS gel electrophoresis, followed by PVDF membrane transfer. After blocking with 5% non-fat milk in TBST, the membranes were incubated with primary antibodies at 4 °C overnight and then incubated with horseradish peroxidase-conjugated secondary antibodies for 1 h at room temperature. The expression of protein was detected and quantified using a chemiluminescent imaging and analysis system (Bio-Rad, Hercules, CA, USA).

## *1.16* *Lentivirus infection*

The negative control (NC) and three sh-Gbp5 lentiviruses were constructed by Genechem Co., Ltd. (Shanghai, China). Treg cells were sorted and seeded in a six-well plate at about 60% density for 24 h before infection. The 200 µL lentivirus at a dose of 100 MOI (1 × 10^8^ TU/mL) and 40 µL HiTranG A infection enhancement reagents were added to each well to infect Treg cells. After 14-16 h of incubation, the medium was replaced with Treg cell differentiation medium for continue culturing. After 48 h infection, puromycin at 3 µg/mL was used to kill non-infected cells and only resistant clones were grown until all cells in the control group died. The efficacy of Gbp5 silencing was determined by Western blotting in the Treg cell lysate for subsequent experiments.

## *1.17 Mitochondrial respiration analysis*

The oxidative phosphorylation function (OXPHOS) of Treg cell was determined by mitochondrial oxygen consumption on a Seahorse XFe24 high-resolution respirometry (Seahorse Bioscience, Billerica, MA, USA) as reported previously . The cells were grown on Seahorse XFe24 cell culture plate (Seahorse Bioscience) coated by anti-CD3 and poly-l-lysine, and cultured in expansion medium. After treatment with ginsenoside Ro for 48 h, the media was then exchanged with Seahorse XF DMEM media contained with 2 mM glutaMAX (Invitrogen), 1 mM sodium pyruvate (Invitrogen), and 10 mM glucose, which were equilibrated for 30 min at 37 °C before the experiment. The baseline oxygen consumption rate (OCR), maximal respiration capacity (MRC), spare respiratory capacity (SPC) and ATP production OCR (ATP-pro) were recorded by continuous injections with mitochondrial respiratory chain inhibitors during continuous oxygen measurements with three cycles of mixing (150 sec), waiting (120 sec), and measuring (210 sec).

## *1.18 Mito-Tracker and Calcein/PI staining*

Treg cells were grown on glass coverslips coated by anti-CD3 and poly-l-lysine, and cultured in expansion medium. After supplement with ginsenoside Ro, the cells were incubated with Mito-Tracker Green (100 nM, C1048, Beyotime) and Lyso-Tracker Red (1:10000, C1046, Beyotime) probes for 40 min at 37 °C in the dark. For pyroptosis staining, the cells were stained with Calcein and PI probes (1:1000, C2015, Beyotime) for 40 min at 37 °C in the dark after treatment with loganic acid in 20 ng/mL LPS incubation. The nucleus of living cells was stained with Hoechst 33258 (C1011, Beyotime) for 10 min. The images were acquired using a STELLARIS 5 confocal laser scanning microscope system (Leica).

## *1.19* *Quantification and statistical analysis*

The *in vivo* and *in vitro* experimental groups were designed to establish equal size, blinding and randomization. All group sizes represent the numbers of experimental independent values, and these independent values were used to evaluate statistical analyses. Statistical analyses were undertaken for the experiments where each group sizes (n) ≥ 3 replicates in *in vitro* experiments, and (n) ≥ 5 biological replicates in *in vivo* experiments. The results are expressed as mean ± SD in *in vitro* study and mean ± SEM in *in vivo* study. Between-group comparisons were done with the unpaired, two-tailed Student’s t test and multiple groups were analyzed using one-way ANOVA followed by a Tukey’s post hoc test. The post hoc tests were conducted only if the F in ANOVA achieved the necessary statistical significance level and there was no significant variance inhomogeneity. The analyses were performed using GraphPad Prism 8.0 (Boston, MA, USA) and Differences with *P* < 0.05 was taken to indicate statistically significant.

## *1.20 Data availability*

The data of this study supporting the findings are available from the corresponding authors through reasonable request.

#
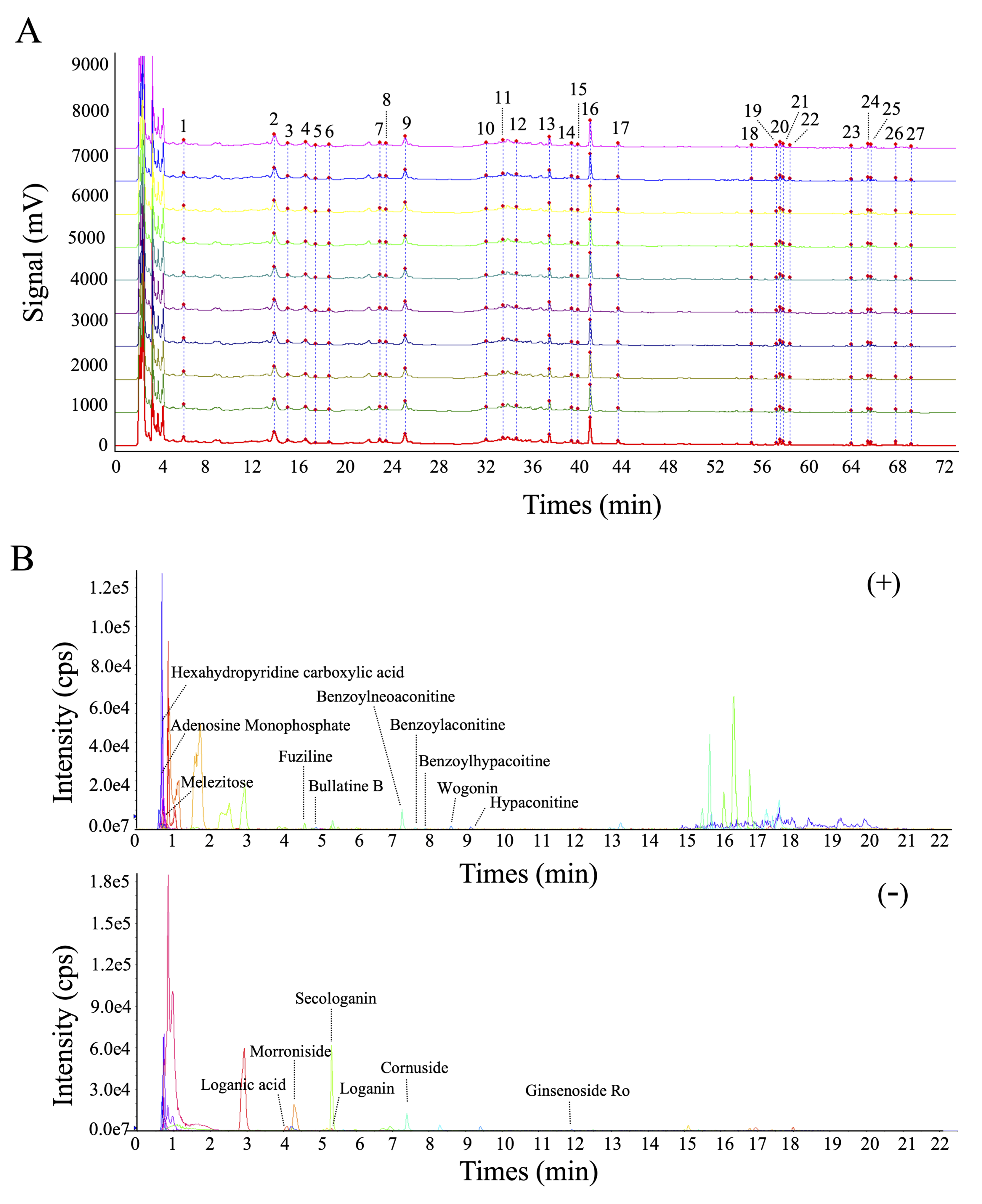
2 Supplementary Figures

**Figure S1 Quality control and chemical component analysis of SFSY.** **(A)** The fingerprint analysis of ten batches extracts by high-performance liquid chromatograph. **(B)** The prototype compounds of SFSY in plasma after intragastric administration of SFSY were characterized using LC-Q-TOF-MS.


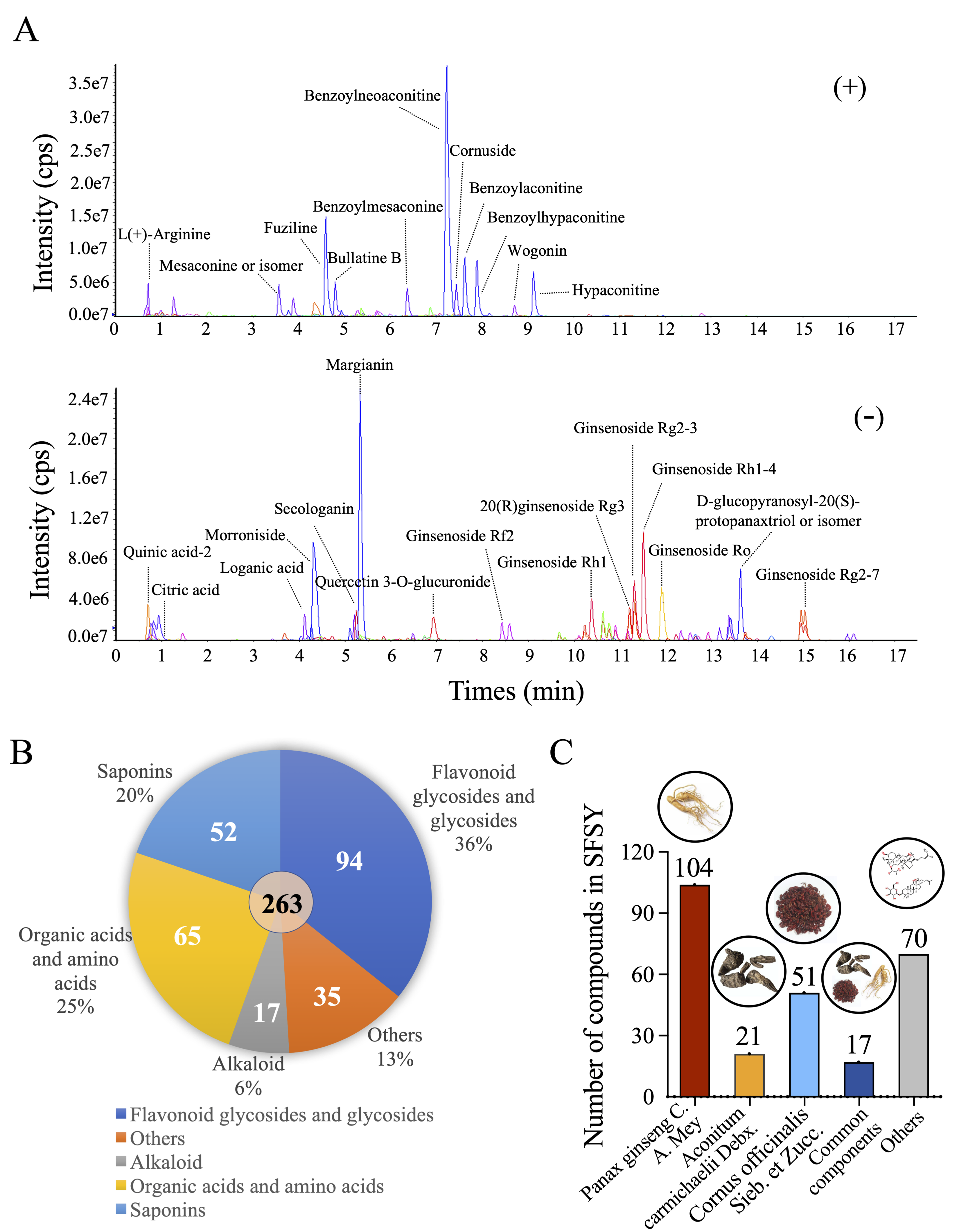


**Figure S2 Chemical identification of SFSY using liquid chromatography with LC-Q-TOF-MS. (A)** The chemical base peak ion chromatogram of SFSY in the negative ion mode and positive ion mode. **(B)** Structural classification of prototype compounds contained in SFSY. (C) The number of chemical components for each herb.


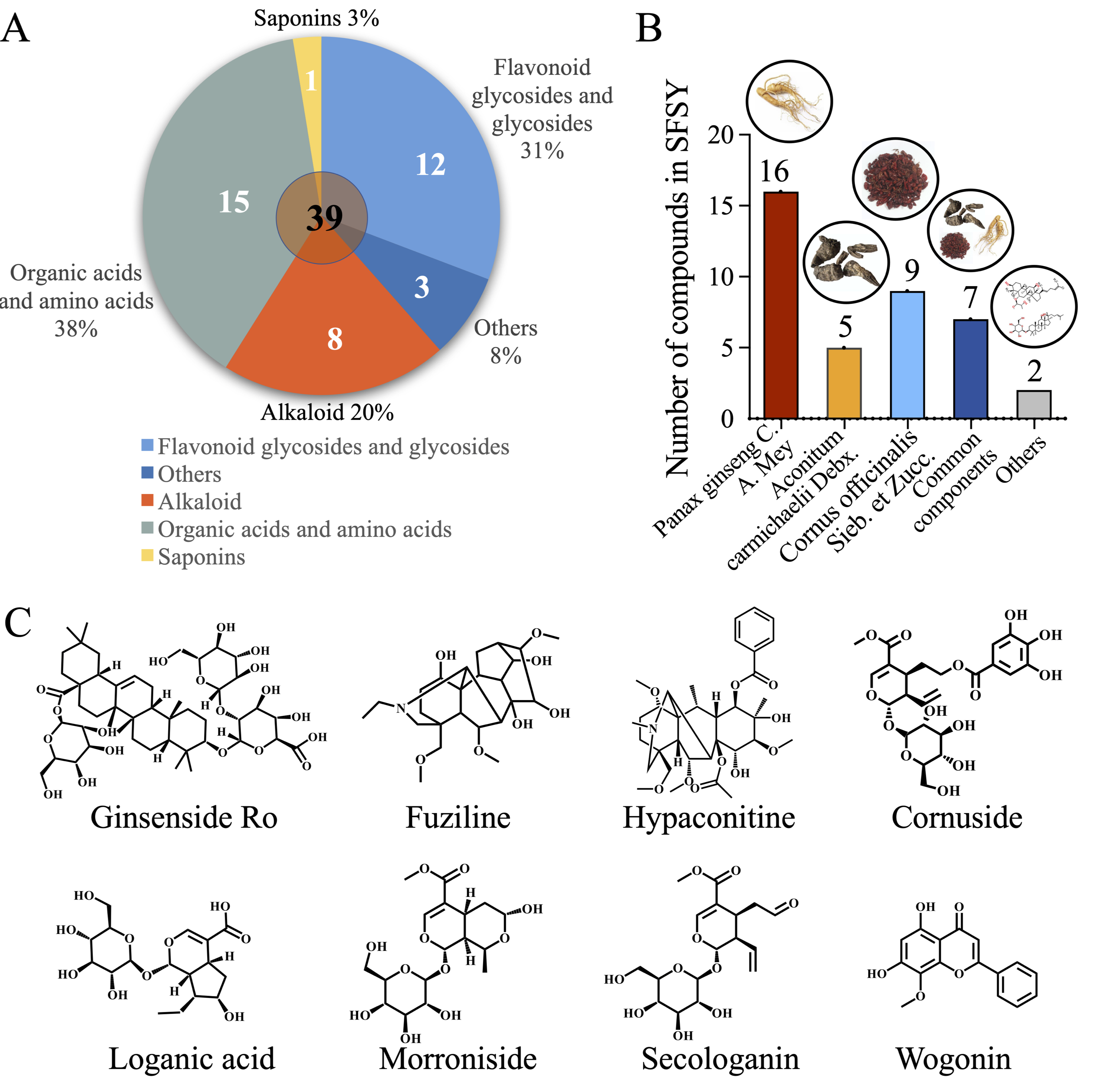


**Figure S3 The chemical compounds with prototype structures in plasma after intragastric administration of SFSY using the LC-Q-TOF-MS system. (A)** Structural classification of prototype chemical compounds in plasma. **(B)** The number of prototype chemical compounds in plasma for each herb. **(C)** Eight structures of the main chemical compounds in plasma.

**Figure S4 SFSY treatment enhanced Th cell function in *in vivo*. (A)** HS/R-induced rat injury model was performed by withdrawing blood from the right femoral arteries until the MAP reached 30-40 mmHg, which was maintained for 2 h and then resuscitation for 2 or 4 h. The rats in the HS /R + SFSY group were intragastrically administered twice with SFSY (1.25-5 g/kg/day) prior to HS and resuscitation, respectively. **(B)** Flow cytometry gate strategy for Th cells and Ts cells in PBMCs and lungs. **(C-D)** The frequency of Th and Ts cells in PBMCs and lungs was detected and analyzed by flow cytometry. Compared with Sham group, ^***^*P* < 0.001; compared with HS/R group, ^#^*P* < 0.05, ^##^*P* < 0.01 and ^###^*P* < 0.001.

**Figure S5 SFSY treatment enhanced Treg cell function following HS/R induction.** **(A)** Flow cytometry gate strategy for Treg cells in PBMCs and lungs. **(B)** The Treg cell function in lungs were analyzed by immunofluorescence staining and co-localization analysis. Compared with Sham group, ^*^*P* < 0.05; compared with HS/R group, ^#^*P* < 0.05.

**Figure S6 SFSY treatment enhanced Treg cell function in *in vitor.* (A)** The protocol of isolating and culturing naïve splenic CD4^+^CD25^+^ Treg cells under Treg cell polarizing conditions in the presence or absence of SFSY. **(B)** The Treg cell function were analyzed by immunofluorescence staining. Scale bars = 50 µm.

**Figure S7 SFSY mitigated HS/R-induced metabolic disorders and lymphocyte depletion.** **(A)** The heart rate was continuously monitored by a digital biological signal acquisition and processing system. **(B-C)** After resuscitation 4 h, the arterial blood was collected into anticoagulant tube with heparin lithium, then the blood gas and electrolyte were analyzed by a VetStat ™ analyzer. **(D)** After 4 h of resuscitation, the complete blood count was detected by a hematology analyzer. **(E)** The effect of SFSY on the lymphocyte percentage was tested by flow cytometry after HS/R for 4 h. Compared with Sham group, ^*^*P* < 0.05, ^**^*P* < 0.01 and ^***^*P* < 0.001; compared with HS/R group, ^#^*P* < 0.05 and ^##^*P* < 0.01.

**Figure S8 The effects of SFSY treatment on HS/R (4 h)-induced metabolic disorder. (A-B)** After resuscitation, the arterial blood was collected from aorta and the levels of blood gas and electrolyte were performed by a VetStat ™ analyzer. **(C-D)** The complete blood count was detected by a hematology analyzer after the arterial blood was collected into anticoagulant tube with EDTA-2K. Compared with Sham group, ^*^*P* < 0.05 and ^**^*P* < 0.001; compared with HS/R group, ^#^*P* < 0.05, and ^##^*P* < 0.01.

**Figure S9 SFSY treatment mitigated HS/R (2 h)-induced metabolic disorders and lymphocyte depletion. (A-B)** After resuscitation 2 h, the arterial blood was collected into anticoagulant tube with heparin lithium, then the blood gas and electrolyte were analyzed by a VetStat ™ analyzer. **(C-D)** After 2 h of resuscitation, the complete blood count was detected by a hematology analyzer. **(E)** The effect of SFSY on the lymphocyte percentage was tested by flow cytometry after HS/R for 2 h. Compared with Sham group, ^*^*P* < 0.05, and ^**^P < 0.01; compared with HS/R group, ^#^*P* < 0.05.

**Figure S10 SFSY pretreatment mitigated HS/R (2 h)- or HS/R (4 h)-induced multiple-organ dysfunction. (A)** The hemotoxylin and eosin staining were used to analyze the HS/R (4 h)-induced histopathological changes of lungs, livers and kidneys. Scale bars = 50 µm or 200 µm. **(B)** The HS/R (2 h)-induced histopathological changes of lungs were examined by H&E staining. Scale bars = 100 µm.

**Figure S11 SFSY mitigated HS/R-induced multiple-organ dysfunction. (A)** The LDH (lactate dehydrogenase) concentration in serum were analyzed by biochemical method. **(B)** The microcirculation in the intestine was measured by a laser speckle imaging system before the rat had been sacrificed. Compared with Sham group, ^*^*P* < 0.05; compared with HS/R group, ^#^*P* < 0.05.

**Figure S12 Multiple Treg-activator pathways were regulated by SFSY treatment. (A)** The mRNA abundance analyses of each sample. **(B)** The dispersion analyses showed significant correlation in mRNA expression among the Sham group, HS/R group, and HS/R + SFSY group. **(C)** Volcano plot showed the transcriptional changes identified in Sham group *vs* HS/R group**. (D)** Annotated GO biological processes were assigned in differentially expressed genes.

**Figure S13** **SFSY inhibited innate immune pathways in CD4^+^CD25^+^Treg cells. (A) T**he Venn diagram analysis identified the differential genes between Sham group *vs* HS/R group with HS/R group vs HS/R + SFSY group. **(B-C)** SFSY significantly impeded the HS/R-induced activation of chemokine receptor, C-type lectin receptor, NOD-like receptor, and cytokine-cytokine receptor interaction pathways in Treg cells. Compared with Sham group, ^*^*P* < 0.05, ^**^*P* < 0.01 and ^***^*P* < 0.001; compared with HS/R group, ^#^*P* < 0.05, ^##^*P* < 0.01 and ^###^*P* < 0.001.

**Figure S14 SFSY** **inhibited innate immune pathways in lungs. (A-C)** The mRNA expression of key genes in the four immune pathways were validated by qPCR. Compared with Sham group, ^*^*P* < 0.05, ^**^*P* < 0.01 and ^***^*P* < 0.001; compared with HS/R group, ^#^*P* < 0.05, ^##^*P* < 0.01 and ^###^*P* < 0.001.

**Figure S15 The effect of SFSY on innate immune pathways was related to Treg cell function.** **(A-D)** The association between differential genes and FOXP3 expression in lungs were analyzed by correlation coefficient plots.

**Figure S16 The effect of the main eight prototype components on inflammation in LPS-induced A549 cell model.** **(A-B)** The mRNA expression of inflammation-related was analize by qPCR assay. Compared with ctrl group, ^*^*P* < 0.05, ^**^*P* < 0.01 and ^***^*P* < 0.001; compared with LPS group, ^#^*P* < 0.05, ^##^*P* < 0.01 and ^###^*P* < 0.001.

**Figure S17 The effect of the main prototype components on enhancing Treg cell function is Stat1-, CXCL10- and Gbp5-dependent. (A)** The qPCR assay was used to analyze the effects of ginsenoside Ro, hypaconitine, loganic acid, secologanin or wogonin treatment on the mRNA expressions of Stat1, Ebi3, CXCL10 and Gbp5 in A549 and Treg cells. **(B-C)** The co-localization of FOXP3 and DAPI was analyzed in Treg cells by immunofluorescence and confocal microscope. Scale bars = 5 µm or 50 µm. Compared with ctrl group, ^*^*P* < 0.05, ^**^*P* < 0.01 and ^***^*P* < 0.001; compared with LPS group, ^#^*P* < 0.05.

**Figure S18 Ginsenoside Ro and hypaconitine enhanced Treg cell function in a Stat1-dependent manner. (A)** The co-localization of FOXP3 and DAPI in Treg cells was analyzed by immunofluorescence staining. Scale bars = 50 µm. **(B)** The mitochondrial content of Treg cells was analyzed after pretreating with ginsenoside Ro or/and Fludar. Scale bars = 10 µm. **(C)** The baseline oxygen consumption rate (Basal OCR), maximal respiration capacity (MRC), ATP production OCR (ATP-pro) and spare respiratory capacity (SPC) were recorded and analyzed by a Seahorse XFe24 high-resolution respirometry. ^*^*P* < 0.05, ^**^*P* < 0.01 and ^***^*P* < 0.001.


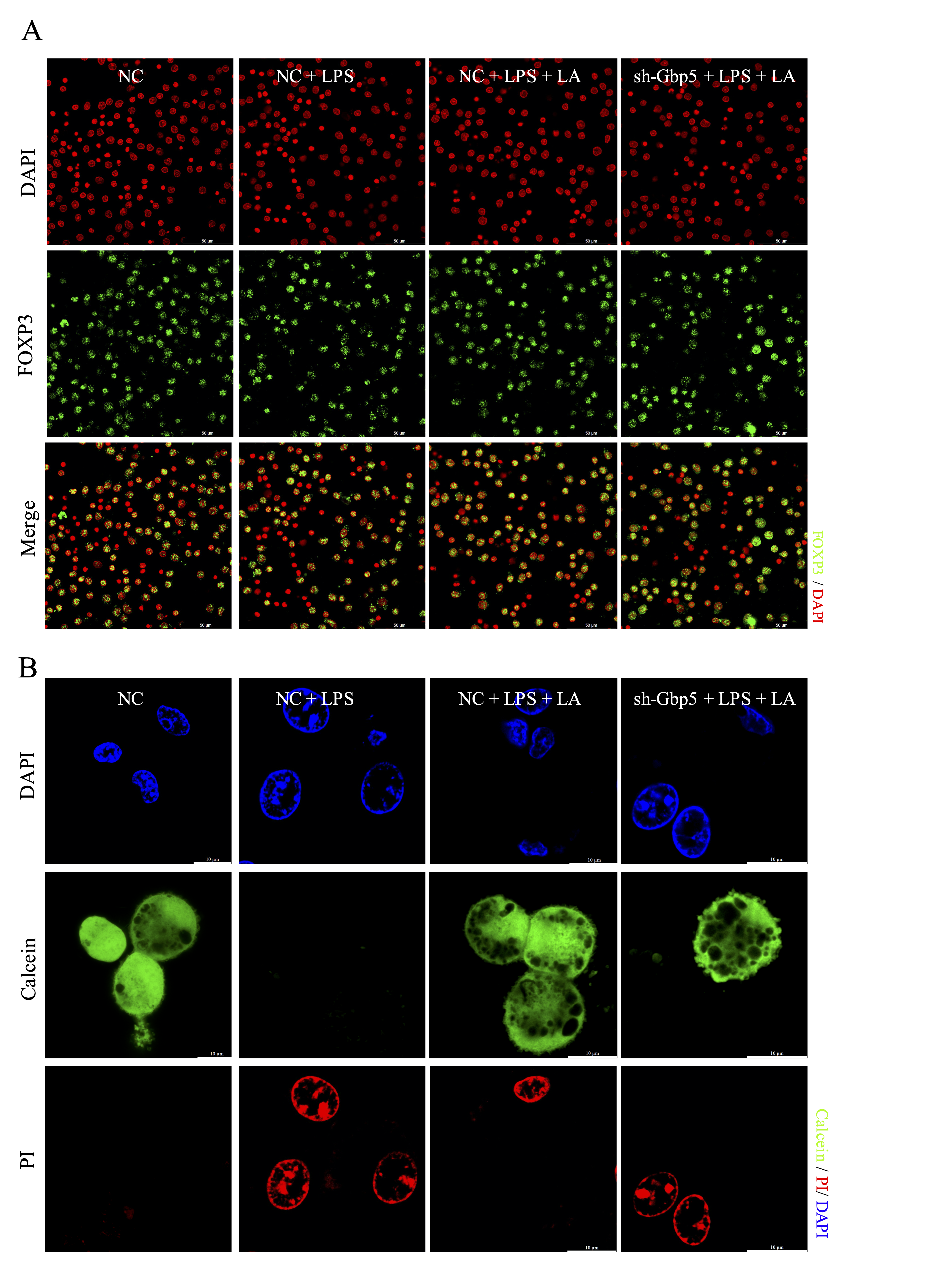


**Figure S19 Loganic acid inhibited pyroptosis of Treg cell in a Gbp5-dependent manner. (A)** The immunofluorescence staining was used to analyze the co-localization of FOXP3 and DAPI in Treg cells. Scale bars = 50 µm. **(B)** The pyroptosis of Treg cell was evaluated using a calcein/PI staining kit. Scale bars = 10 µm.


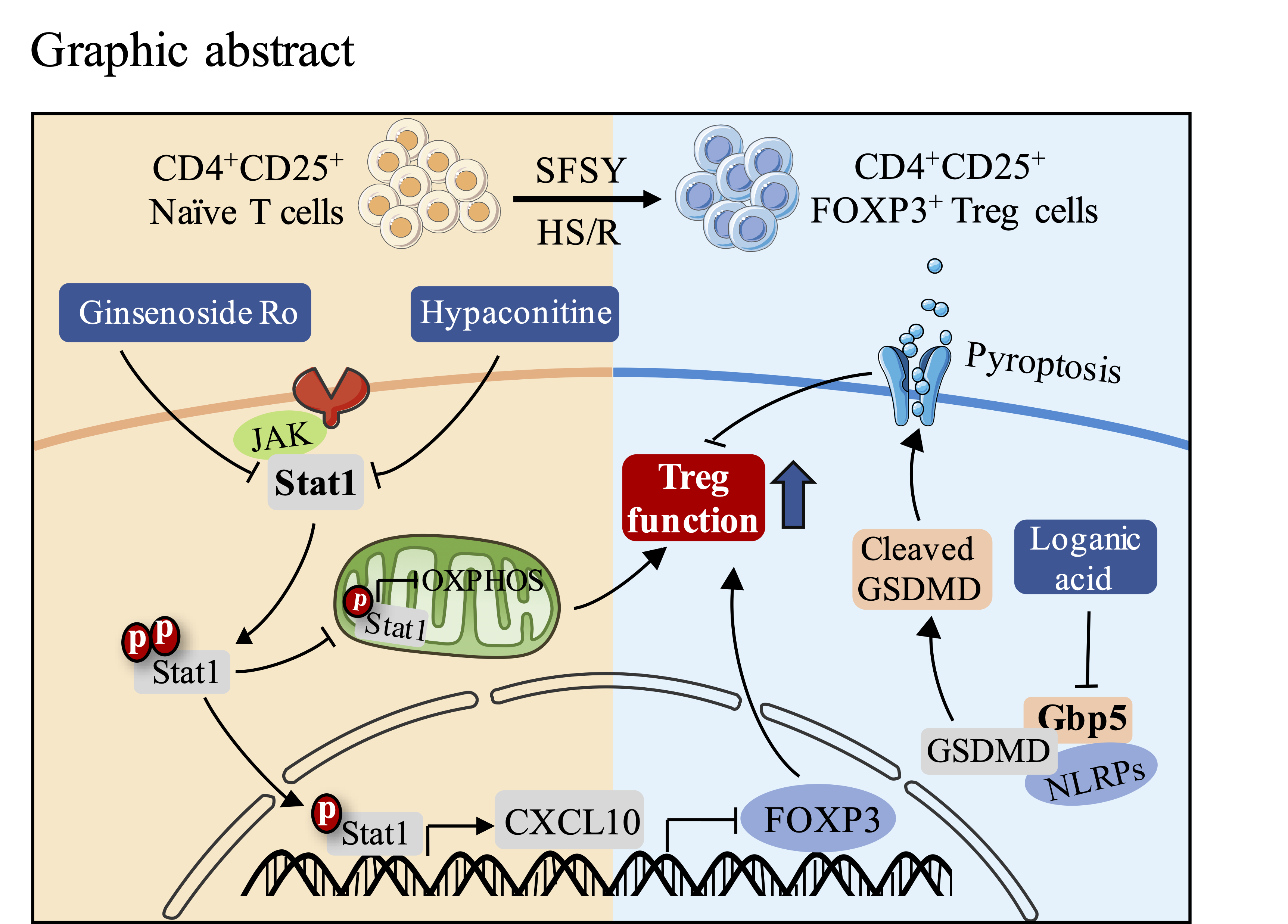


**Graphic abstract.** The study revealed the multi-component and multi-target mechanism of SFSY against HS/R injury that ginsenoside Ro, hypaconitine and loganic acid enhanced Treg cell function via Stat1- and Gbp5-dependent Foxp3 induction. Ginsenoside Ro circumvented the translocation of phosphorylated Stat1 to mitochondria, thereby increasing mitochondrial function of Treg cells, hypaconitine inhibited the phosphorylation of Stat1, then reduced CXCL10 transcription and promoted FOXP3 expression, and loganic acid mitigated the activation of Gbp5 to inhibit Treg cell pyroptosis mediated by GSDMD cleavage.

# 3. Supplementary Tables

**Table S1 The information of the mai**n components in SFSY identified by LC-MS/MS analysis

| **No.** | **Component name** | **Observed RT (min)** | **Formula** | **Adduct/ Charge** | **Area** | **Found Mass** | **Mass Error (ppm)** |
| --- | --- | --- | --- | --- | --- | --- | --- |
| 1 | Loganin | 5.33 | C17H26O10.HCOOH | [M-H]- | 1.01E+08 | 435.1505 | -0.7 |
| 2 | Monoside | 4.33 | C17H26O11.HCOOH | [M-H]- | 6.20E+07 | 451.1454 | -0.6 |
| 3 | D-glucopyranosyl-20(S)-protopanaxtriol | 13.63 | C37H62O10 | [M-H]- | 2.91E+07 | 665.4259 | -1.7 |
| 4 | Cornus officinalis glycoside | 7.44 | C24H30O14 | [M+NH4]+ | 1.81E+07 | 560.1972 | -0.3 |
| 5 | Secologanin or isomer | 5.2 | C17H24O10 | [M+FA-H]- | 8.00E+06 | 433.1348 | -0.9 |
| 6 | Loganin-2 | 4.26 | C17H26O10.HCOOH | [M-H]- | 4.87E+06 | 435.1502 | -1.3 |
| 7 | Perseitol Heptaacetate | 5.74 | C21H30O14 | [M+Na]+ | 4.45E+06 | 529.1525 | -0.5 |
| 8 | Dangyaoside | 5.28 | C16H22O9 | [M+H]+ | 4.06E+06 | 359.1336 | -0.2 |
| 9 | 5-hydroxymethylfurfural-1 | 2.04 | C6H6O3 | [M+H]+ | 3.48E+06 | 127.0387 | -1.8 |
| 10 | Hyperoside | 6.75 | C21H20O12 | [M-H]- | 3.02E+06 | 463.0872 | -2.1 |
| 11 | Bamboo joint ginseng saponin-2 | 12.64 | C42H66O14 | [M-H]- | 2.97E+06 | 793.4363 | -2.1 |
| 12 | Tongguanteng glycoside | 14.3 | C42H66O14 | [M-H]- | 2.71E+06 | 793.4364 | -2 |
| 13 | 4-Methylumbelliferone-2 | 4.36 | C10H8O3 | [M+H]+ | 2.68E+06 | 177.0543 | -1.9 |
| 14 | 1,7-di-O-galloyl-Dsedoheptulose | 1.58 | C14H18O11 | [M+Na]+ | 2.54E+06 | 385.0739 | -0.7 |
| 15 | 1,2,3,6-tetragalloylglucose | 6.11 | C34H28O22 | [M-H]- | 1.98E+06 | 787.0985 | -1.9 |
| 16 | D-mannose D- (+) | 0.72 | C6H12O6 | [M-H]- | 1.93E+06 | 179.0558 | -1.8 |
| 17 | Quercetin-2 | 6.97 | C15H10O7 | [M+H]+ | 1.54E+06 | 303.0496 | -1.1 |
| 18 | Melibiose | 0.71 | C12H22O11 | [M+Na]+ | 1.31E+06 | 365.1048 | -1.8 |
| 19 | Mangiferin-2 | 5.28 | C19H18O11 | [M-H]- | 1.02E+06 | 421.0768 | -1.9 |
| 20 | 1,3,6-tri-O-galloylglucose-3 | 4.58 | C27H24O18 | [M-H]- | 9.74E+05 | 635.0879 | -1.7 |
| 21 | 1,3,6-tri-O-galloylglucose-1 | 4.88 | C27H24O18 | [M-H]- | 9.33E+05 | 635.0878 | -1.8 |
| 22 | 6-Methylcoumarin-2 | 5.37 | C10H8O2 | [M+H]+ | 9.12E+05 | 161.0594 | -2.1 |
| 23 | Quercetin-3 | 8.77 | C15H10O7 | [M+H]+ | 9.08E+05 | 303.0495 | -1.6 |
| 24 | Quercetin-1 | 6.77 | C15H10O7 | [M+H]+ | 8.38E+05 | 303.0495 | -1.5 |
| 25 | Forsythia glycoside-1 | 6.6 | C29H36O15 | [M-H]- | 6.83E+05 | 623.1968 | -2.2 |
| 26 | Kaempferol-3-O-Rutinoside-2 | 6.99 | C27H30O15 | [M-H]- | 6.53E+05 | 593.1498 | -2.3 |
| 27 | Naringenin-7-O-glucoside-3 | 7.09 | C21H22O10 | [M-H]- | 6.15E+05 | 433.1129 | -2.5 |
| 28 | 4-Methylumbelliferone-1 | 5.23 | C10H8O3 | [M+H]+ | 5.85E+05 | 177.0542 | -2.2 |
| 29 | 1,3,6-tri-O-galloylglucose-2 | 5.23 | C27H24O18 | [M-H]- | 5.80E+05 | 635.0881 | -1.5 |
| 30 | Gallic acid-4-O-β-D-glucoside or isomer | 1.16 | C13H16O10 | [M-H]- | 5.42E+05 | 331.0667 | -1.2 |
| 31 | 7S-O-morroniside or isomer | 4.36 | C17H26O11 | [M+Na]+ | 5.40E+05 | 429.1364 | -0.8 |
| 32 | Korilajing-3 | 1.42 | C27H22O18 | [M-H]- | 5.37E+05 | 633.0729 | -0.7 |
| 33 | Melezitose | 0.73 | C18H32O16 | [M+Na]+ | 5.17E+05 | 527.1579 | -0.7 |
| 34 | Adenosine Monophosphate | 0.98 | C10H14N5O7P | [M+H]+ | 5.09E+05 | 348.0701 | -0.8 |
| 35 | Camphor | 7.38 | C10H16O | [M+H]+ | 4.71E+05 | 153.127 | -2.3 |
| 36 | Paeoniflorin | 5.69 | C23H28O11.HCOOH | [M-H]- | 4.29E+05 | 525.1606 | -1.5 |
| 37 | Luteolin-1 | 7.26 | C15H10O6 | [M+H]+ | 3.72E+05 | 287.0542 | -2.7 |
| 38 | Beta-penta-O-galloyl-glucose | 6.87 | C41H32O26 | [M-H]- | 3.72E+05 | 939.1093 | -1.7 |
| 39 | Zhimu saponin | 9.55 | C45H76O19.HCOOH | [M-H]- | 3.63E+05 | 965.4942 | -2.1 |
| 40 | Pineolin monomethyl ether-D-glucoside | 6.92 | C26H32O11 | [M-H]- | 3.61E+05 | 519.1859 | -2.5 |
| 41 | Naringin-4 | 7.13 | C15H12O5 | [M+H]+ | 3.56E+05 | 273.0752 | -2.1 |
| 42 | Korilajing-2 | 2.1 | C27H22O18 | [M-H]- | 3.47E+05 | 633.073 | -0.5 |
| 43 | Astragaloside | 7.26 | C21H20O11 | [M+H]+ | 3.41E+05 | 449.1071 | -1.6 |
| 44 | Rutoside | 6.69 | C27H30O16 | [M+Na]+ | 3.39E+05 | 633.1419 | -1.1 |
| 45 | 6-Methylcoumarin-1 | 4.52 | C10H8O2 | [M+H]+ | 2.53E+05 | 161.0593 | -2.7 |
| 46 | Naringenin-7-O-glucoside-2 | 6.19 | C21H22O10 | [M-H]- | 2.52E+05 | 433.1128 | -2.7 |
| 47 | Root bark glycoside | 7.56 | C21H24O10 | [M-H]- | 2.52E+05 | 435.1284 | -2.9 |
| 48 | Forsythia glycoside-2 | 6.18 | C29H36O15 | [M-H]- | 2.49E+05 | 623.1965 | -2.6 |
| 49 | Hesperidin | 7.31 | C28H34O15 | [M-H]- | 2.47E+05 | 609.1811 | -2.4 |
| 50 | Naringin-3 | 9.2 | C15H12O5 | [M+H]+ | 2.46E+05 | 273.0754 | -1.4 |
| 51 | Isorhamnetin-3-O-glucoside | 7.53 | C22H22O12 | [M-H]- | 2.45E+05 | 477.1027 | -2.3 |
| 52 | Myricetin | 7.61 | C15H10O8 | [M-H]- | 2.31E+05 | 317.0296 | -2.2 |
| 53 | Dihydrodaidzein | 8.29 | C15H10O4 | [M-H]- | 2.29E+05 | 253.0498 | -3.1 |
| 54 | Isoimperatorin | 12.64 | C16H14O4 | [M-H]- | 2.27E+05 | 269.0811 | -3 |
| 55 | 8-epigallocatechin or isomer | 5.15 | C17H26O10 | [M+NH4]+ | 2.27E+05 | 408.1859 | -1.2 |
| 56 | D-Fructose-6-phosphate disodium salt hydrate | 0.75 | C6H13O9P | [M-H]- | 2.18E+05 | 259.0218 | -2.7 |
| 57 | 5-Hydroxymethylfurfural-2 | 0.76 | C6H6O3 | [M+H]+ | 1.86E+05 | 127.0386 | -3.1 |
| 58 | Naringenin-7-O-glucoside-1 | 7.85 | C21H22O10 | [M-H]- | 1.59E+05 | 433.1126 | -3.3 |
| 59 | Harpagoside | 9.38 | C24H30O11 | [M+Na]+ | 1.53E+05 | 517.1676 | -0.9 |
| 60 | Liquiritin | 6.4 | C21H22O9 | [M-H]- | 1.51E+05 | 417.1181 | -2.4 |
| 61 | Spiraeoside-2 | 7.87 | C21H20O12 | [M-H]- | 1.50E+05 | 463.0867 | -3.3 |
| 62 | Arabinofuranosyluracil | 1 | C9H12N2O6 | [M-H]- | 1.47E+05 | 243.0618 | -2.1 |
| 63 | Luteolin-2 | 9.77 | C15H10O6 | [M+H]+ | 1.30E+05 | 287.0547 | -1.2 |
| 64 | Naringenin-2 | 6.43 | C15H12O5 | [M+H]+ | 1.26E+05 | 273.0754 | -1.2 |
| 65 | Luteolin-5 | 7.49 | C15H10O6 | [M+H]+ | 1.26E+05 | 287.0545 | -1.6 |
| 66 | Tigogenin | 11.32 | C27H44O3 | [M+H]+ | 1.22E+05 | 417.3354 | -2.1 |
| 67 | Petunidin-3-O-beta-glucopyranoside | 7.55 | C22H23O12 | [M]+ | 1.14E+05 | 479.1182 | -0.5 |
| 68 | Luteolin-4 | 7.08 | C15H10O6 | [M+H]+ | 1.12E+05 | 287.0546 | -1.4 |
| 69 | Corilagin | 3.12 | C27H22O18 | [M-H]- | 1.09E+05 | 633.0722 | -1.8 |
| 70 | Pedunculoside-2 | 11.44 | C36H58O10.HCOOH | [M-H]- | 1.07E+05 | 695.3992 | -2.9 |
| 71 | Salsoline | 1.86 | C11H15NO2 | [M+H]+ | 1.06E+05 | 194.1171 | -2.4 |
| 72 | Puerarin | 5.2 | C21H20O9 | [M+H]+ | 1.03E+05 | 417.1176 | -1.1 |
| 73 | Kaempferol 3-O-Rutinoside-1 | 7.23 | C27H30O15 | [M-H]- | 9.81E+04 | 593.1496 | -2.7 |
| 74 | Hexahydroxybiphenyl dicarbonyl-Dglucoside | 3.64 | C27H22O18 | [M-H]- | 9.79E+04 | 633.0723 | -1.6 |
| 75 | 1-Methyladenosine | 2.48 | C11H15N5O4 | [M+H]+ | 9.45E+04 | 282.119 | -2.5 |
| 76 | Eriodictyol-2 | 8.14 | C15H12O6 | [M-H]- | 9.28E+04 | 287.055 | -3.8 |
| 77 | Plantamajoside | 5.79 | C29H36O16 | [M-H]- | 8.21E+04 | 639.1928 | -0.4 |
| 78 | Naringenin-1 | 7.89 | C15H12O5 | [M+H]+ | 7.93E+04 | 273.0757 | -0.1 |
| 79 | Eriodictyol-1 | 7.3 | C15H12O6 | [M-H]- | 7.39E+04 | 287.0552 | -3.1 |
| 80 | Pedunculoside-1 | 10.45 | C36H58O10.HCOOH | [M-H]- | 7.36E+04 | 695.3985 | -4 |
| 81 | Wogonin | 8.77 | C16H12O5 | [M+H]+ | 7.32E+04 | 285.0754 | -1.1 |
| 82 | Esculin | 3.92 | C15H16O9 | [M-H]- | 7.11E+04 | 339.0711 | -3.2 |
| 83 | Hesperetin | 7.33 | C16H14O6 | [M+H]+ | 6.84E+04 | 303.0861 | -0.8 |
| 84 | Formononetin | 10.46 | C16H12O4 | [M-H]- | 5.86E+04 | 267.0655 | -2.8 |
| 85 | Isoliquiritigenin | 8.22 | C15H12O4 | [M+H]+ | 5.73E+04 | 257.0805 | -1.2 |
| 86 | Luteolin-3 | 6.45 | C15H10O6 | [M+H]+ | 5.61E+04 | 287.0545 | -2 |
| 87 | Syringetin-3-O-galactoside | 7.55 | C23H24O13 | [M-H]- | 5.52E+04 | 507.1128 | -3.1 |
| 88 | Liquiritigenin | 8.19 | C15H12O4 | [M-H]- | 5.48E+04 | 255.0651 | -4.6 |
| 89 | Pedunculoside-3 | 10.86 | C36H58O10.HCOOH | [M-H]- | 4.92E+04 | 695.3989 | -3.3 |
| 90 | Nobiletin | 11.92 | C21H22O8 | [M+H]+ | 4.89E+04 | 403.138 | -1.9 |
| 91 | Apigenin | 9.73 | C15H10O5 | [M-H]- | 3.80E+04 | 269.0446 | -3.4 |
| 92 | Isorhamnetin | 9.95 | C16H12O7 | [M-H]- | 3.78E+04 | 315.0505 | -1.8 |
| 93 | Scutellarin | 7.55 | C21H18O12 | [M+H]+ | 3.39E+04 | 463.087 | -0.3 |
| 94 | Mangiferin-1 | 7.07 | C19H18O11 | [M-H]- | 3.30E+04 | 421.0762 | -3.3 |
| 95 | L (+)-Arginine | 0.71 | C6H14N4O2 | [M+H]+ | 1.56E+07 | 175.1188 | -0.9 |
| 96 | Citric Acid | 0.93 | C6H8O7 | [M-H]- | 1.46E+07 | 191.0199 | 0.8 |
| 97 | Quercetin 3-O-glucuronide | 6.93 | C21H18O13 | [M-H]- | 1.45E+07 | 477.0669 | -1.2 |
| 98 | Quininic acid-2 | 0.7 | C7H12O6 | [M-H]- | 1.38E+07 | 191.056 | -0.5 |
| 99 | L-Malic acid | 0.78 | C4H6O5 | [M-H]- | 1.21E+07 | 133.0143 | 0.8 |
| 100 | loganic acid | 4.16 | C16H24O10 | [M-H]- | 8.90E+06 | 375.1295 | -0.5 |
| 101 | p-Coumalic acid-3 | 5.18 | C9H8O3 | [M-H]- | 6.73E+06 | 163.0399 | -1 |
| 102 | L-Pyroglutamic acid | 0.99 | C5H7NO3 | [M+H]+ | 5.37E+06 | 130.0496 | -1.8 |
| 103 | Maleic acid | 0.78 | C4H4O4 | [M-H]- | 4.68E+06 | 115.0037 | 0.3 |
| 104 | Gallic acid | 1.45 | C7H6O5 | [M-H]- | 3.37E+06 | 169.0144 | 0.6 |
| 105 | Caffeic acid-3 | 4.48 | C9H8O4 | [M-H]- | 2.82E+06 | 179.0349 | -0.5 |
| 106 | Ellagic acid | 6.73 | C14H6O8 | [M-H]- | 2.70E+06 | 300.9984 | -2 |
| 107 | Caffeic acid-1 | 4.72 | C9H8O4 | [M-H]- | 2.35E+06 | 179.0348 | -0.9 |
| 108 | Asiatic acid | 13.41 | C30H48O5 | [M-H]- | 1.97E+06 | 487.3414 | -3 |
| 109 | Tyrosine | 1.02 | C9H11NO3 | [M+H]+ | 1.91E+06 | 182.0809 | -1.6 |
| 110 | 2-3-ethenyl-5-methoxycarbonyl-2 | 5.84 | C17H24O11 | [M-H]- | 1.58E+06 | 403.1243 | -0.7 |
| 111 | 2-Hydroxycinnamic acid | 1.02 | C9H8O3 | [M+H]+ | 1.18E+06 | 165.0543 | -2 |
| 112 | Phenylalanine | 1.76 | C9H11NO2 | [M+H]+ | 1.16E+06 | 166.086 | -1.4 |
| 113 | Leucine | 1.12 | C6H13NO2 | [M+H]+ | 1.16E+06 | 132.1016 | -2.5 |
| 114 | Caffeic acid-2 | 3.77 | C9H8O4 | [M-H]- | 1.13E+06 | 179.0348 | -1.3 |
| 115 | p-Coumalic acid-4 | 5.83 | C9H8O3 | [M-H]- | 1.11E+06 | 163.0399 | -0.8 |
| 116 | Quercetin-3-O-β-D-glucuronic acid or isomer | 6.48 | C21H18O13 | [M-H]- | 1.04E+06 | 477.0667 | -1.5 |
| 117 | Syringic Acid | 5.48 | C9H10O5 | [M-H]- | 9.82E+05 | 197.0453 | -1.1 |
| 118 | Piscidic Acid | 2.57 | C11H12O7 | [M-H]- | 7.90E+05 | 255.0508 | -1.1 |
| 119 | Proline | 0.72 | C5H9NO2 | [M+H]+ | 7.82E+05 | 116.07 | -4.8 |
| 120 | Coniferic acid-2 | 5.6 | C10H10O4 | [M-H]- | 7.59E+05 | 193.0503 | -1.6 |
| 121 | L-Tyrosine | 4.75 | C9H11NO3 | [M+H]+ | 6.83E+05 | 182.0808 | -1.9 |
| 122 | 3-O-Feruloylquinic acid | 5.7 | C17H20O9 | [M-H]- | 6.78E+05 | 367.1026 | -2.4 |
| 123 | 4-Coumaric acid-1 | 5.88 | C9H8O3 | [M+H]+ | 6.60E+05 | 165.0544 | -1.3 |
| 124 | (Z)-9,12,13-trihydroxyoctadec-15-enoic acid | 11.16 | C18H34O5 | [M+Na]+ | 6.59E+05 | 353.23 | 0.4 |
| 125 | Quininic acid-1 | 5.35 | C7H12O6 | [M-H]- | 6.35E+05 | 191.0559 | -1.3 |
| 126 | Pipecolic acid | 0.77 | C6H11NO2 | [M+H]+ | 4.86E+05 | 130.0862 | -0.5 |
| 127 | Sodium pantothenate-2 | 2.55 | C9H17NO5 | [M+H]+ | 4.58E+05 | 220.1176 | -1.5 |
| 128 | Fat-soluble vitamin | 0.94 | C6H5NO2 | [M+H]+ | 4.32E+05 | 124.0388 | -4.1 |
| 129 | Chlorogenic acid-1 | 4.48 | C16H18O9 | [M-H]- | 4.28E+05 | 353.0871 | -1.9 |
| 130 | p-Coumalic acid-1 | 4.39 | C9H8O3 | [M-H]- | 4.23E+05 | 163.0397 | -2.1 |
| 131 | Coniferic acid-1 | 4.33 | C10H10O4 | [M-H]- | 4.14E+05 | 193.0501 | -2.6 |
| 132 | Azelaic acid | 7.52 | C9H16O4 | [M-H]- | 3.43E+05 | 187.0971 | -2.5 |
| 133 | Mucic acid | 0.73 | C6H10O8 | [M-H]- | 3.39E+05 | 209.0298 | -2.2 |
| 134 | P-Coumalic acid-2 | 3.83 | C9H8O3 | [M-H]- | 2.86E+05 | 163.0398 | -1.5 |
| 135 | Isoferulic acid | 6.29 | C10H10O4 | [M-H]- | 2.65E+05 | 193.0502 | -2.4 |
| 136 | 3-Indoleacetic acid | 2.41 | C10H9NO2 | [M+H]+ | 2.23E+05 | 176.0704 | -1.4 |
| 137 | Tryptophan | 2.94 | C11H12N2O2 | [M-H]- | 2.14E+05 | 203.0822 | -1.8 |
| 138 | Protocatechuic acid-3 | 13.18 | C18H18O2 | [M-H]- | 2.13E+05 | 265.1228 | -2.3 |
| 139 | Catechol | 2.74 | C6H6O2 | [M-H]- | 1.65E+05 | 109.0293 | -1.8 |
| 140 | Sodium pantothenate-1 | 3.05 | C9H17NO5 | [M+H]+ | 1.63E+05 | 220.1177 | -1.1 |
| 141 | Protocatechuic acid-1 | 2.74 | C7H6O4 | [M-H]- | 1.63E+05 | 153.0192 | -1.1 |
| 142 | Sinapic acid | 5.88 | C11H12O5 | [M+H]+ | 1.54E+05 | 225.0753 | -2.1 |
| 143 | Sodium 4-hydroxy-benzoate | 6.75 | C7H6O3 | [M+H]+ | 1.50E+05 | 139.0385 | -3.3 |
| 144 | A-hexahydrocyclopenta pyran-4-carboxylic acid | 6.37 | C16H24O9 | [M-H]- | 1.46E+05 | 359.1339 | -2.5 |
| 145 | 3-Phenyllactic acid | 5.71 | C9H10O3 | [M-H]- | 1.42E+05 | 165.0554 | -1.7 |
| 146 | Salicylic acid-2 | 6.9 | C7H6O3 | [M-H]- | 1.27E+05 | 137.0241 | -2.4 |
| 147 | 4-Coumaric acid-2 | 1.05 | C9H8O3 | [M+H]+ | 1.21E+05 | 165.0545 | -1 |
| 148 | Abscisic acid | 8.34 | C15H20O4 | [M-H]- | 1.15E+05 | 263.128 | -3.2 |
| 149 | Protocatechuic acid-2 | 3.61 | C7H6O3 | [M-H]- | 1.15E+05 | 137.0242 | -1.7 |
| 150 | Oxybenzoic acid | 8.35 | C23H30O12 | [M-H]- | 1.14E+05 | 497.1653 | -2.3 |
| 151 | Chlorogenic acid-2 | 5.07 | C16H18O9 | [M-H]- | 1.08E+05 | 353.0871 | -2 |
| 152 | SINAPIC ACID | 4.74 | C11H12O5 | [M-H]- | 1.07E+05 | 223.0605 | -2.9 |
| 153 | Succinic Acid | 1.12 | C4H6O4 | [M-H]- | 1.04E+05 | 117.0189 | -3.8 |
| 154 | 9-(2,3-dihydroxypropoxy)-9-oxononanoic acid | 7.12 | C12H22O6 | [M-H]- | 1.03E+05 | 261.1338 | -2.3 |
| 155 | Oxan-2-yl oxycyclopentyl acetic acid | 8.06 | C18H30O8 | [M-H]- | 8.96E+04 | 373.1855 | -3.3 |
| 156 | Oxyphenyl prop-2-enoic acid | 6.24 | C16H20O9 | [M-H]- | 8.81E+04 | 355.1022 | -3.4 |
| 157 | N-acetyltryptophan | 6.32 | C13H14N2O3 | [M-H]- | 5.74E+04 | 245.0924 | -3.1 |
| 158 | Salicylic acid-1 | 1.93 | C7H6O3 | [M-H]- | 3.88E+04 | 137.0241 | -2.6 |
| 159 | Ursolic Acid-1 | 17.43 | C30H48O3 | [M+H]+ | 2.38E+04 | 457.3673 | -0.6 |
| 160 | Ginsenoside Rh1-4 | 11.51 | C36H62O9.HCOOH | [M-H]- | 4.82E+07 | 683.436 | -2.3 |
| 161 | Ginsenoside Ro | 11.92 | C48H76O19 | [M-H]- | 3.03E+07 | 955.4885 | -2.4 |
| 162 | Ginsenoside Rh1-3 | 10.38 | C36H62O9.HCOOH | [M-H]- | 1.91E+07 | 683.436 | -2.3 |
| 163 | Ginsenoside Rg2-3 | 11.32 | C42H72O13 | [M-H]- | 1.61E+07 | 783.4875 | -3.3 |
| 164 | 20(R)-ginsenoside Rg3-2 | 11.2 | C42H72O13 | [M+FA-H]- | 1.38E+07 | 829.4932 | -2.8 |
| 165 | Pseudoginsenoside F11-1 | 10.63 | C42H72O14 | [M-H]- | 1.15E+07 | 799.4829 | -2.5 |
| 166 | 20(R)-ginsenoside Rg3-2 | 14.95 | C42H72O13 | [M-H]- | 1.05E+07 | 783.4881 | -2.5 |
| 167 | Adenosine | 1.27 | C10H13N5O4 | [M+H]+ | 9.79E+06 | 268.104 | -0.2 |
| 168 | Ginsenoside F4 or isomer | 13.38 | C42H70O12 | [M+FA-H]- | 9.58E+06 | 811.4832 | -2.1 |
| 169 | Pseudoginsenoside F11-2 | 10.76 | C42H72O14 | [M-H]- | 7.94E+06 | 799.4831 | -2.3 |
| 170 | Ginsenoside Rg2-2 | 10.23 | C42H72O13.HCOOH | [M-H]- | 7.87E+06 | 829.4935 | -2.4 |
| 171 | Ginsenoside Rg2-7 | 14.95 | C42H72O13.HCOOH | [M-H]- | 7.43E+06 | 829.4934 | -2.5 |
| 172 | 20(R)-ginsenoside Rf2 or isomer-2 | 8.59 | C42H74O14 | [M+FA-H]- | 7.16E+06 | 847.5045 | -1.9 |
| 173 | Ginsenoside F2 | 15.04 | C42H72O13.HCOOH | [M-H]- | 6.87E+06 | 829.4931 | -2.9 |
| 174 | 20(R)-ginsenoside Rf2 or isomer-1 | 8.42 | C42H74O14 | [M+FA-H]- | 6.48E+06 | 847.504 | -2.4 |
| 175 | 20(R)-ginsenoside Rg3-1 | 13.74 | C42H72O13 | [M-H]- | 4.51E+06 | 783.4882 | -2.3 |
| 176 | Ginsenoside Rg2-4 | 10.23 | C42H72O13 | [M-H]- | 4.45E+06 | 783.4882 | -2.3 |
| 177 | 20(R)-notoginsenoside R2-2 | 10.897 | C41H70O13 | [M+FA-H]- | 4.33E+06 | 815.478 | -2.3 |
| 178 | Ginsenoside F5 | 11.17 | C41H70O13 | [M-H]- | 4.22E+06 | 769.4726 | -2.3 |
| 179 | Pseudoginsenoside F11-0 | 9.67 | C42H72O14 | [M-H]- | 3.42E+06 | 799.4833 | -2.1 |
| 180 | Ginsenoside Rg5 or isomer | 12.92 | C42H70O13 | [M+FA-H]- | 3.33E+06 | 827.4779 | -2.4 |
| 181 | Ginsenoside Rg1-1 | 9.68 | C42H72O14.HCOOH | [M-H]- | 3.23E+06 | 845.4887 | -2 |
| 182 | Ginsenoside Rh1-1 | 12.22 | C36H62O9.HCOOH | [M-H]- | 2.55E+06 | 683.4358 | -2.6 |
| 183 | 20(R)-notoginsenoside R2-1 | 10.1 | C41H70O13 | [M+FA-H]- | 2.20E+06 | 815.4782 | -2 |
| 184 | Ginsenoside Rg2-1 | 13.74 | C42H72O13.HCOOH | [M-H]- | 1.74E+06 | 829.493 | -3 |
| 185 | Ginsenoside Rh1-2 | 12.65 | C36H62O9.HCOOH | [M-H]- | 1.67E+06 | 683.436 | -2.4 |
| 186 | Panaxydol | 12.79 | C17H24O2 | [M+H]+ | 1.60E+06 | 261.1845 | -1.7 |
| 187 | Pseudoginsenoside F11-3 | 11.42 | C42H72O14 | [M-H]- | 1.02E+06 | 799.4826 | -3 |
| 188 | Ginsenoside Rg2-6 | 12.04 | C42H72O13.HCOOH | [M-H]- | 9.70E+05 | 829.4931 | -2.9 |
| 189 | Guanosine | 1.29 | C10H13N5O5 | [M+H]+ | 8.08E+05 | 284.0989 | -0.3 |
| 190 | Ginsenoside Rg2-5 | 12.05 | C42H72O13 | [M-H]- | 7.33E+05 | 783.4877 | -2.9 |
| 191 | Ginsenoside Rg1-2 | 11.45 | C42H72O14.HCOOH | [M-H]- | 6.81E+05 | 845.4878 | -3.1 |
| 192 | Ginsenoside Rh1 | 11.51 | C36H62O9 | [M-H]- | 5.99E+05 | 637.4299 | -3.4 |
| 193 | Pseudoginsenoside RT5 | 10.72 | C36H62O10.HCOOH | [M-H]- | 4.58E+05 | 699.4305 | -2.8 |
| 194 | Pseudoginsenoside F11-5 | 12.95 | C42H72O14 | [M-H]- | 3.64E+05 | 799.4826 | -2.9 |
| 195 | Albiflorin | 5.36 | C23H28O11 | [M+H]+ | 3.37E+05 | 481.1695 | -1.9 |
| 196 | Pseudoginsenoside F11-4 | 11.85 | C42H72O14 | [M-H]- | 2.93E+05 | 799.4821 | -3.6 |
| 197 | Pseudoginsenoside-3 | 10.75 | C36H62O10 | [M+H]+ | 2.49E+05 | 655.4406 | -1.5 |
| 198 | Panaxadiol-2 | 13.77 | C30H52O3 | [M+H]+ | 2.09E+05 | 461.3984 | -1.2 |
| 199 | Panaxadiol-1 | 12.37 | C30H52O3 | [M+H]+ | 1.77E+05 | 461.3983 | -1.4 |
| 200 | Pseudoginsenoside-2 | 9.79 | C36H62O10 | [M+H]+ | 1.53E+05 | 655.4408 | -1.1 |
| 201 | Notoginsenoside Ft1 | 14.53 | C47H80O17H.COOH | [M-H]- | 1.48E+05 | 961.5349 | -3 |
| 202 | Pedunculoside | 10.89 | C36H58O10.NH3 | [M+H]+ | 1.48E+05 | 668.4362 | -0.9 |
| 203 | Rhoifolin | 4.97 | C27H30O14 | [M+H]+ | 1.36E+05 | 579.1703 | -0.9 |
| 204 | Amygdalin | 6.09 | C20H27NO11.NH3 | [M+H]+ | 1.26E+05 | 475.1932 | 2.1 |
| 205 | Ginsenoside-2 | 14.64 | C48H82O18.HCOOH | [M-H]- | 1.12E+05 | 991.5456 | -2.7 |
| 206 | Nodakenin | 6.97 | C20H24O9 | [M+H]+ | 9.05E+04 | 409.1487 | -1.4 |
| 207 | Ginsenoside-3 | 11.09 | C48H82O18.HCOOH | [M-H]- | 8.73E+04 | 991.546 | -2.3 |
| 208 | Benzoylpaeoniflorin | 9.61 | C30H32O12.NH3 | [M+H]+ | 7.71E+04 | 602.2224 | -1.3 |
| 209 | Pseudoginsenoside-1 | 7.4 | C36H62O10 | [M+H]+ | 7.10E+04 | 655.442 | 0.7 |
| 210 | Araloside A | 10.83 | C42H66O14 | [M-H]- | 5.08E+04 | 793.4354 | -3.2 |
| 211 | Ginsenoside-1 | 14.03 | C48H82O18.HCOOH | [M-H]- | 5.00E+04 | 991.545 | -3.3 |
| 212 | Choline | 0.71 | C5H14NO | [M]+ | 3.44E+06 | 104.1066 | -3.4 |
| 213 | Methyl 3,4,5-trihydroxybenzoate | 4.57 | C20H20O14 | [M-H]- | 1.85E+06 | 483.0771 | -1.9 |
| 214 | DIPALNDKWHCLEA-UHFFFAOYSA-N-1 | 7.37 | C16H28O7 | [M+Na]+ | 1.53E+06 | 355.1722 | -1.4 |
| 215 | DVWKMCPPEMUHBE-RAGYRXETSA-N | 7.08 | C26H34O11 | [M+NH4]+ | 1.34E+06 | 540.2435 | -0.8 |
| 216 | Adenine-2 | 1.27 | C5H5N5 | [M+H]+ | 1.01E+06 | 136.0616 | -1.5 |
| 217 | AHYOMNWKYGMYMB-CIEFDVMPSA-N | 6.37 | C26H34O11 | [M-H]- | 9.64E+05 | 521.2017 | -2.2 |
| 218 | BFCZPWYLRHFBNO-DTJAAUDXSA-N | 1.31 | C12H20O8 | [M+Na]+ | 9.46E+05 | 315.1047 | -1.2 |
| 219 | LBRPLJCNRZUXLS-YTMAOMSOSA-N | 10.33 | C26H30N2O8 | [M+H]+ | 9.25E+05 | 499.2073 | -0.4 |
| 220 | PNYQFRZBMVRYFC-CGWYSGAGSA-N | 6.79 | C22H20O13 | [M-H]- | 9.14E+05 | 491.0819 | -2.5 |
| 221 | Adenine-1 | 0.8 | C5H5N5 | [M+H]+ | 6.64E+05 | 136.0616 | -1.4 |
| 222 | Glutathione (oxidized form) | 0.92 | C20H32N6O12S2 | [M-H]- | 6.37E+05 | 611.1441 | -0.9 |
| 223 | YNMFDPCLPIMRFD-PEXUZNNCSA-N | 6.23 | C26H28O16 | [M-H]- | 5.67E+05 | 595.1292 | -2.2 |
| 224 | LEEYYHJQGXOXHR-MYZNJVEUSA-N-1 | 10.842 | C54H86O24 | [M-H]- | 5.55E+05 | 1117.5407 | -2.6 |
| 225 | RFFYIBOJHUSIGD-PHDUQKSESA-N | 8.89 | C21H36O10 | [M+NH4]+ | 5.40E+05 | 466.264 | -1.4 |
| 226 | QZMAEZWZCGBZFK-VPQYALDZSA-N-1 | 11.75 | C48H76O19 | [M-H]- | 5.19E+05 | 955.4883 | -2.6 |
| 227 | DIPALNDKWHCLEA-UHFFFAOYSA-N-2 | 6.64 | C16H28O7 | [M+Na]+ | 4.59E+05 | 355.1723 | -1.3 |
| 228 | 6-(hydroxymethyl) oxan-2-yl oxypropan | 4.89 | C15H20O8 | [M-H]- | 4.06E+05 | 327.1078 | -2.3 |
| 229 | KBDOXLBZVFQTMH-RXRQAHPVSA-N | 8.93 | C24H30O12 | [M+Na]+ | 3.22E+05 | 533.1624 | -1 |
| 230 | LEEYYHJQGXOXHR-MYZNJVEUSA-N-2 | 11.63 | C54H86O24 | [M-H]- | 2.91E+05 | 1117.5401 | -3.1 |
| 231 | 2-hydroxyquinoline | 2.36 | C9H7NO | [M+H]+ | 2.84E+05 | 146.0597 | -2.4 |
| 232 | Oxyoxan-2-yl methyl 3,4,5-trihydroxybenzoate | 3.3 | C20H20O14 | [M-H]- | 2.59E+05 | 483.0772 | -1.6 |
| 233 | Riboflavin | 5.37 | C17H20N4O6 | [M+H]+ | 2.40E+05 | 377.1451 | -1.3 |
| 234 | 6-(hydroxymethyl) oxan-2-yl oxypropan | 4.11 | C15H20O8 | [M-H]- | 1.90E+05 | 327.1079 | -2 |
| 235 | 6-(hydroxymethyl) oxan-3-yl trihydroxybenzoate | 7.02 | C28H24O16 | [M-H]- | 1.74E+05 | 615.0976 | -2.6 |
| 236 | OVMSOCFBDVBLFW-ZZMVMVDNSA-N | 8.67 | C31H38O11 | [M+Na]+ | 1.68E+05 | 609.2298 | -1.4 |
| 237 | RWEVZSYWIIZZFV-UHFFFAOYSA-N | 7.58 | C26H34O11 | [M+Na]+ | 1.33E+05 | 545.1983 | -2 |
| 238 | 5-ethenyl-3-hydroxy-4-(3-hydroxyprop-1-en-2-yl) | 6.09 | C23H32O9 | [M+Na]+ | 1.25E+05 | 475.1933 | -1.2 |
| 239 | LIVNOUBJFOXZOR-YMILTQATSA-N | 4.57 | C16H22O9 | [M+NH4]+ | 1.04E+05 | 376.16 | -0.5 |
| 240 | IFIQVSCCFRXSJV-ZIZFEDMCSA-N | 10.31 | C30H48O6 | [M-H]- | 1.04E+05 | 503.3361 | -3.4 |
| 241 | IUCHKMAZAWJNBJ-DKAZIEIMSA-N | 15.318 | C36H56O9 | [M-H]- | 5.02E+04 | 631.3831 | -3.2 |
| 242 | YVMUBJPWZKUGBC-NUTNSJPXSA-N | 9.91 | C22H30O11 | [M+Na]+ | 4.63E+04 | 493.1695 | 3 |
| 243 | IFIQVSCCFRXSJV-ZIZFEDMCSA-N | 11.4 | C30H48O6 | [M-H]- | 4.42E+04 | 503.3356 | -4.4 |
| 244 | PUETUDUXMCLALY-UHFFFAOYSA-N-2 | 6.17 | C20H26O6 | [M-H]- | 4.30E+04 | 361.1642 | -4 |
| 245 | 3,4,5-trihydroxy-6-(hydroxymethyl) oxan-2-yl] | 8.19 | C36H58O12 | [M+Na]+ | 4.22E+04 | 705.3814 | -1 |
| 246 | PUETUDUXMCLALY-UHFFFAOYSA-N-1 | 7.65 | C20H26O6 | [M-H]- | 3.92E+04 | 361.1643 | -3.7 |
| 247 | Benzoylmesaconine | 7.23 | C31H43NO10 | [M+H]+ | 2.00E+08 | 590.2956 | -0.6 |
| 248 | Fuziline | 4.59 | C24H39NO7 | [M+H]+ | 6.20E+07 | 454.28 | 0.1 |
| 249 | Benzoylaconitine | 7.62 | C32H45NO10 | [M+H]+ | 3.53E+07 | 604.3112 | -0.8 |
| 250 | Benzoylhypaconine | 7.89 | C31H43NO9 | [M+H]+ | 3.41E+07 | 574.3009 | -0.2 |
| 251 | Hypaconitine | 9.12 | C33H45NO10 | [M+H]+ | 2.72E+07 | 616.3113 | -0.4 |
| 252 | Bullatine B or isomer | 4.79 | C24H39NO6 | [M+H]+ | 1.96E+07 | 438.2849 | -0.3 |
| 253 | Mesaconine or isomer | 3.56 | C24H39NO9 | [M+H]+ | 1.70E+07 | 486.2697 | 0 |
| 254 | Benzoylmesaconine or isomer | 6.37 | C31H43NO11 | [M+H]+ | 1.57E+07 | 606.2907 | -0.3 |
| 255 | Songorine or isomer | 3.88 | C22H31NO3 | [M+H]+ | 9.97E+06 | 358.2375 | -0.6 |
| 256 | Mesaconitine | 8.71 | C33H45NO11 | [M+H]+ | 6.03E+06 | 632.306 | -0.9 |
| 257 | Chasmanine | 5.7 | C25H41NO6 | [M+H]+ | 2.84E+06 | 452.3003 | -0.9 |
| 258 | Sn-Glycero-3-phosphocholine | 0.73 | C8H21NO6P | [M]+ | 1.33E+06 | 258.1094 | -2.5 |
| 259 | Senbusine C or isomer | 4.4 | C24H39NO7 | [M+FA-H]- | 1.28E+06 | 498.2698 | -2 |
| 260 | Tetrahydroberberine | 7.49 | C20H17NO4 | [M+H]+ | 1.18E+06 | 336.1226 | -1.4 |
| 261 | Sinapoylcholine | 4.53 | C16H24NO5 | [M]+ | 2.51E+05 | 310.1644 | -1.7 |
| 262 | Indole-3-aldehyde | 2.97 | C9H7NO | [M+H]+ | 1.80E+05 | 146.0598 | -1.8 |
| 263 | 10-OH benzoylaconine or isomer | 6.76 | C32H45NO11 | [M+H]+ | 1.72E+05 | 620.3057 | -1.3 |

**Table S2**. The information of the main components in plasma identified by LC-MS/MS analysis

| **NO.** | **Component name** | **Observed RT (min)** | **Formula** | **Adduct/Charge** | **Area** | **Found Mass** | **Mass Error (ppm)** |
| --- | --- | --- | --- | --- | --- | --- | --- |
| 1 | D-mannose D-(+) | 0.67 | C6H12O6 | [M-H]- | 7.21E+04 | 179.0556 | -2.7 |
| 2 | Melibiose | 0.68 | C12H22O11 | [M+Na]+ | 2.14E+05 | 365.1053 | -0.4 |
| 3 | Adenosine Monophosphate | 0.72 | C10H14N5O7P | [M+H]+ | 3.04E+04 | 348.0706 | 0.6 |
| 4 | Melezitose | 0.73 | C18H32O16 | [M+Na]+ | 2.80E+04 | 527.1582 | -0.1 |
| 5 | D-Fructose-6-phosphate disodium salt hydrate | 0.75 | C6H13O9P | [M-H]- | 7.08E+04 | 259.0212 | -4.6 |
| 6 | Morroniside | 4.31 | C17H26O11.HCOOH | [M-H]- | 1.36E+05 | 451.1452 | -1.1 |
| 7 | Secologanin or isomer | 5.2 | C17H24O10 | [M+FA-H]- | 7.83E+03 | 433.1342 | -2.1 |
| 8 | Loganetin | 5.32 | C17H26O10.HCOOH | [M-H]- | 2.02E+05 | 435.1501 | -1.5 |
| 9 | Cornuside | 7.38 | C24H30O14 | [M-H]- | 6.32E+04 | 541.1557 | -1.1 |
| 10 | Dihydrodaidzein | 8.29 | C15H10O4 | [M-H]- | 1.91E+04 | 253.05 | -2.5 |
| 11 | Wogonin | 8.55 | C16H12O5 | [M+H]+ | 6.52E+03 | 285.0751 | -2.3 |
| 12 | Apigenin | 9.41 | C15H10O5 | [M-H]- | 1.48E+04 | 269.0449 | -2.4 |
| 13 | Choline | 0.67 | C5H14NO | [M]+ | 2.98E+05 | 104.1065 | -4.3 |
| 14 | Glutathione (oxidized form) | 0.73 | C20H32N6O12S2 | [M-H]- | 2.46E+05 | 611.1434 | -2.1 |
| 15 | Riboflavin | 5.33 | C17H20N4O6 | [M+H]+ | 1.58E+04 | 377.1455 | -0.1 |
| 16 | Sn-Glycero-3-phosphocholine | 0.71 | C8H21NO6P | [M]+ | 8.07E+04 | 258.1098 | -1 |
| 17 | Indole-3-aldehyde | 2.92 | C9H7NO | [M+H]+ | 1.64E+05 | 146.0598 | -1.8 |
| 18 | Fuziline | 4.52 | C24H39NO7 | [M+H]+ | 9.40E+03 | 454.28 | 0.1 |
| 19 | Bullatine B or isomer | 4.77 | C24H39NO6 | [M+H]+ | 2.23E+03 | 438.2839 | -2.6 |
| 20 | Benzoylmesaconine | 7.17 | C31H43NO10 | [M+H]+ | 3.14E+04 | 590.296 | 0 |
| 21 | Benzoylaconitine | 7.54 | C32H45NO10 | [M+H]+ | 2.37E+03 | 604.3119 | 0.4 |
| 22 | Benzoylhypaconine | 7.82 | C31H43NO9 | [M+H]+ | 2.14E+03 | 574.3007 | -0.7 |
| 23 | Hypaconitine | 9.09 | C33H45NO10 | [M+H]+ | 4.31E+03 | 616.3114 | -0.4 |
| 24 | Pipecolic acid | 0.59 | C6H11NO2 | [M+H]+ | 5.18E+04 | 130.0857 | -4.4 |
| 25 | Proline | 0.7 | C5H9NO2 | [M+H]+ | 1.41E+05 | 116.0701 | -4.3 |
| 26 | Quinic acid-2 | 0.71 | C7H12O6 | [M-H]- | 1.45E+05 | 191.0554 | -3.7 |
| 27 | L-Malic acid | 0.74 | C4H6O5 | [M-H]- | 3.89E+04 | 133.014 | -1.7 |
| 28 | 2-Hydroxycinnamic acid | 0.84 | C9H8O3 | [M+H]+ | 1.73E+05 | 165.0545 | -0.9 |
| 29 | Tyrosine | 0.84 | C9H11NO3 | [M+H]+ | 2.30E+05 | 182.081 | -1.1 |
| 30 | Citric Acid | 0.85 | C6H8O7 | [M-H]- | 1.76E+06 | 191.0197 | -0.2 |
| 31 | Leucine | 0.87 | C6H13NO2 | [M+H]+ | 5.27E+05 | 132.1016 | -2.4 |
| 32 | Phenylalanine | 1.68 | C9H11NO2 | [M+H]+ | 6.52E+05 | 166.0861 | -1 |
| 33 | Sodium pantothenate-2 | 2.47 | C9H17NO5 | [M+H]+ | 1.86E+05 | 220.1178 | -0.8 |
| 34 | Tryptophan | 2.91 | C11H12N2O2 | [M-H]- | 4.51E+05 | 203.0824 | -0.9 |
| 35 | loganic acid | 4.09 | C16H24O10 | [M-H]- | 1.78E+04 | 375.1291 | -1.5 |
| 36 | Ellagic acid | 6.74 | C14H6O8 | [M-H]- | 2.35E+04 | 300.9986 | -1.4 |
| 37 | Salicylic acid | 6.94 | C7H6O3 | [M-H]- | 2.76E+04 | 137.0243 | -1 |
| 38 | 9-(2,3-dihydroxypropoxy)-9-oxononanoic acid | 7.16 | C12H22O6 | [M-H]- | 5.54E+03 | 261.1338 | -2.2 |
| 39 | Ginsenoside Ro | 11.91 | C48H76O19 | [M-H]- | 4.52E+03 | 955.4887 | -2.2 |

**Table S3**. The absolute quantitative results of the protoype compounds of SFSY in plasma

| **Component Name** | **FragmentMass** | **RetentionTime** | **DP** | **CE** | **Area**  **(QC)** | **Area**  **(Sham)** | **Area**  **(HS/R)** | **Area(HS/R+**  **SFSY-1)** | **Area(HS/R+SFSY-2)** |
| --- | --- | --- | --- | --- | --- | --- | --- | --- | --- |
| Ginsenoside Ro | 955.491 | 11.91 | -80 | -20 | 6.49E+04 | 2.16E+04 | 1.62E+04 | 6.22E+04 | 6.06E+04 |
| Phenylalanine | 120.087 | 1.68 | 80 | 40 | 3.38E+07 | 1.98E+07 | 3.20E+07 | 3.17E+07 | 3.49E+07 |
| Proline | 70.069 | 0.7 | 80 | 40 | 8.21E+06 | 5.85E+06 | 8.54E+06 | 8.02E+06 | 8.14E+06 |
| Tryptophan | 116.052 | 2.91 | -80 | -40 | 4.92E+06 | 6.28E+06 | 3.98E+06 | 3.91E+06 | 4.03E+06 |
| Citric acid | 87.012 | 0.85 | -80 | -40 | 4.48E+06 | 2.79E+06 | 4.50E+06 | 4.87E+06 | 4.81E+06 |
| 2-Hydroxycinnamic acid | 95.049 | 0.84 | 80 | 40 | 4.22E+06 | 3.68E+06 | 4.03E+06 | 3.99E+06 | 3.95E+06 |
| Quininic acid-2 | 111.009 | 0.71 | -80 | -40 | 2.84E+06 | 1.62E+06 | 2.70E+06 | 2.88E+06 | 2.88E+06 |
| Salicylic acid-2 | 93.035 | 6.94 | -80 | -40 | 1.96E+06 | 1.16E+06 | 1.96E+06 | 1.99E+06 | 2.01E+06 |
| Leucine | 86.103 | 0.87 | 80 | 40 | 1.34E+06 | 7.08E+05 | 1.35E+06 | 1.18E+06 | 1.24E+06 |
| Tyrosine | 123.047 | 0.84 | 80 | 40 | 1.10E+06 | 9.13E+05 | 9.74E+05 | 9.70E+05 | 1.03E+06 |
| Hexahydropyridine carboxylic acid | 69.982 | 0.59 | 80 | 40 | 4.53E+05 | 3.00E+05 | 5.03E+05 | 4.46E+05 | 4.70E+05 |
| Sodium pantothenate-2 | 90.055 | 2.47 | 80 | 40 | 2.44E+05 | 1.42E+05 | 2.63E+05 | 2.62E+05 | 2.63E+05 |
| Loganic acid | 69.035 | 4.09 | -80 | -40 | 4.66E+04 | N/A | N/A | 8.74E+04 | 8.97E+04 |
| 9-oxononanoic acid | 125.098 | 7.16 | -80 | -40 | 3.50E+04 | 3.49E+04 | 3.26E+04 | 3.81E+04 | 3.83E+04 |
| Tannic acid | 283.997 | 6.74 | -80 | -45 | 2.61E+04 | 4.83E+04 | 4.64E+04 | 3.31E+04 | 2.77E+04 |
| L-malic acid | 78.961 | 0.74 | -80 | -40 | 1.76E+04 | 1.26E+04 | 1.83E+04 | 2.07E+04 | 2.17E+04 |
| Indole-3-aldehyde | 91.053 | 2.92 | 80 | 40 | 5.84E+07 | 7.25E+07 | 4.46E+07 | 4.42E+07 | 4.64E+07 |
| sn-Glycero-3-phosphocholine | 141.07 | 0.71 | 80 | 40 | 1.07E+06 | 6.62E+05 | 1.07E+06 | 1.16E+06 | 1.17E+06 |
| Benzoyl neoaconitine | 540.261 | 7.17 | 80 | 45 | 3.11E+05 | 2.40E+04 | 2.66E+04 | 4.27E+05 | 4.36E+05 |
| Bullatine B or isomer | 438.305 | 4.77 | 80 | 40 | 2.59E+05 | N/A | N/A | 3.99E+05 | 4.10E+05 |
| Fuziling | 436.271 | 4.52 | 80 | 40 | 2.18E+05 | 1.62E+04 | 1.66E+04 | 2.88E+05 | 3.15E+05 |
| Hypaconitine | 556.279 | 9.09 | 80 | 40 | 1.09E+05 | 4.57E+03 | 3.92E+03 | 1.92E+05 | 2.05E+05 |
| Benzoyl diaconitine | 542.276 | 7.82 | 80 | 45 | 9.57E+04 | 1.35E+04 | 1.23E+04 | 9.90E+04 | 9.83E+04 |
| Benzoyl aconitine | 554.274 | 7.54 | 80 | 50 | 3.90E+04 | 5.46E+03 | 5.41E+03 | 4.07E+04 | 4.34E+04 |
| Choline | 58.065 | 0.67 | 80 | 40 | 2.19E+07 | 1.83E+07 | 2.11E+07 | 2.59E+07 | 2.36E+07 |
| Riboflavin | 243.089 | 5.33 | 80 | 40 | 1.05E+06 | 4.97E+05 | 1.15E+06 | 1.15E+06 | 1.19E+06 |
| Glutathione (oxidized form) | 306.076 | 0.73 | -80 | -40 | 9.85E+05 | 3.62E+05 | 4.95E+05 | 1.51E+06 | 1.70E+06 |
| Melezitose | 527.158 | 0.69 | 80 | 40 | 9.21E+05 | 6.48E+04 | 8.13E+05 | 7.74E+05 | 7.68E+05 |
| Adenosine Monophosphate | 136.061 | 0.72 | 80 | 40 | 5.48E+05 | 2.42E+06 | 1.11E+05 | 4.15E+05 | 3.60E+05 |
| D-Fructose-6-phosphate | 78.959 | 0.75 | -80 | -40 | 4.58E+05 | 1.79E+05 | 3.67E+05 | 5.97E+05 | 6.33E+05 |
| Daidzein | 253.051 | 8.29 | -80 | -40 | 2.34E+05 | 1.22E+05 | 2.51E+05 | 2.24E+05 | 2.37E+05 |
| Wogonin | 270.053 | 8.55 | 80 | 40 | 2.09E+05 | 6.68E+04 | 2.39E+05 | 2.22E+05 | 2.39E+05 |
| Apigenin | 133.03 | 9.41 | -80 | -40 | 1.76E+05 | 8.25E+04 | 1.86E+05 | 1.81E+05 | 1.94E+05 |
| Melibiose | 191.04 | 0.68 | 80 | 40 | 1.73E+05 | 1.80E+05 | 1.19E+05 | 1.65E+05 | 1.65E+05 |
| D-mannose D-(+) | 59.014 | 0.67 | -80 | -40 | 1.61E+05 | 9.80E+04 | 1.30E+05 | 1.70E+05 | 1.72E+05 |
| Morroniside | 155 | 4.31 | -80 | -50 | 1.50E+05 | N/A | N/A | 2.71E+05 | 2.52E+05 |
| Cornuside | 169.019 | 7.38 | -80 | -45 | 1.31E+05 | 2.26E+05 | 1.60E+05 | 1.34E+05 | 1.11E+05 |
| Strychnoside | 101.025 | 5.32 | -80 | -40 | 5.86E+04 | N/A | N/A | 1.11E+05 | 1.09E+05 |
| Secologanin | 297.004 | 5.2 | -80 | -40 | 2.88E+04 | N/A | 3.01E+04 | 3.06E+04 | 3.14E+04 |

**Table S4.** Primer list of rat

| **Genes** | **Forward primer** | **Reverse primer** |
| --- | --- | --- |
| Dock2 | ATGACAAGCCTCGCATCTTCTGTG | TGGTGGTGGTGGTGGTGAGTC |
| Stat1 | TCGCACCTTCGTCCTCTTCCAG | TTCACCAACAGTCTCAGCTTCACAG |
| C-type lectin | GGCTCCCACTGCTACTTTACAACTG | GAAATCCTGCTCTTCCTGGCTGTG |
| Irf1 | CCAAGCAGGGAAGGGACCAAAC | TCACACTAACCTCAGAACGCCAATC |
| Ebi3 | AGCCCAGGACCTCACCGATTATG | CAGCCACCCTCAAGTAGTCAACATC |
| Cxcl17 | AAGCACCACAGGAAGTCACAAAGG | AGGGCAGAGTCTCGGAACTATAAGG |
| Tnfrsf9 | TTCTCTGGTTCTCTGTGCCCAAATG | CCTCCTCCTCCTTCTTCTTCCTCTG |
| Csf2rb | GGAGCGAGTGGAGCAATGAGTG | TGAGGATGAGGAAGACCAGGATGAG |
| Cxcr3 | GAGAAGCAGGCAGCACGAGAC | GGCAATGTCCGAGGCATCTAGC |
| Pf4 | CTGCTTCTTCTGGGTCTGCTGTTG | AGGCTGGTGATGCGTTTGAGATG |
| Ccr5 | ATTGTCCTCCTCCTGACCACCTTC | GGCAGCAGTGTGTCATCCCAAG |
| Ccr4 | ACCAAGGAAGGCATCAAGGCATTC | TGAACAGCACCAGAACCACAACAG |
| Ccl2 | CTCACCTGCTGCTACTCATTCACTG | CTTCTTTGGGACACCTGCTGCTG |
| CXCL10 | TGAAAGCGGTGAGCCAAAGAAGG | CTGGGTAAAGGGAGGTGGAGAGAC |
| Card9 | AATACGACAGGCAGCGACAACAC | AAAACCCGCAGCATCCCTTCAC |
| Camp | CCGTGCTGTGGATGACTTCAACC | AGTCTCCTTCACTCGGAACCTCAC |
| Caspase-4 | TTGGGCTATGATGTGGTGGTGAAAG | TGCTGTCTGATGTTTGGTGCTCTG |
| Nlrc4 | GGGCTCTGCTGCTGAAGTTACAC | CCGTGGTGGTGGTGACAATGAC |
| Gbp5 | CTGGGAGATGTGGAGAAGGTTGATG | ACGAAGGTGCTACTGAGGAGGATC |
| Gbp2 | TCAGGGAAGGATACGAGAACGAGAG | TGAAGAGTTTGGCAGAGAGGTTTGG |
| IL-6 | ACTTCCAGCCAGTTGCCTTCTTG | TGGTCTGTTGTGGGTGGTATCCTC |
| IL-1β | AATCTCACAGCAGCATCTCGACAAG | TCCACGGGCAAGACATAGGTAGC |
| TNF-α | CACCACGCTCTTCTGTCTACTGAAC | TGGGCTACGGGCTTGTCACTC |
| IL-10 | CTGCTCTTACTGGCTGGAGTGAAG | TGGGTCTGGCTGACTGGGAAG |
| TGF-β | GGACCGCAACAACGCAATCTATG | TCTGGCACTGCTTCCCGAATG |
| FOXP3 | GAAGAATGCCATCCGCCACAAC | ATTCATCTACGGTCCACACTGCTC |
| Rorc | GCGGCTCTCAGGCTTCATGG | GTGTGGTTGTTGGCATTGTAGGC |
| Gata3 | GTGGCGGCGAGATGGTACTG | GTGGTGGTGGTGGTCTGACAG |
| Tbx21 | GTGAATGACGGTGAGCCAGAGG | TGGTAGGCAGTCACGGCAATG |
| β-Actin | CATTGTCACCAACTGGGACGATA | GGATGGCTACGTACATGGCTG |

**Table S5**. Primer list of Human

| **Genes** | **Forward primer** | **Reverse primer** |
| --- | --- | --- |
| IL-6 | GGTGTTGCCTGCTGCCTTCC | GTTCTGAAGAGGTGAGTGGCTGTC |
| IL-1β | GGACAGGATATGGAGCAACAAGTGG | CAACACGCAGGACAGGTACAGATTC |
| TNF-α | ACCCTCAGACGCCACATCCC | GGAACAAGCACCGCCTGGAG |
| β-Actin | ATCGTGCGTGACATTAAGGAGAAG | AGGAAGGAAGGCTGGAAGAGTG |

**Table S6. Primer list of mitoc**hondrial function

| **Genes** | **Forward primer** | **Reverse primer** |
| --- | --- | --- |
| Drp-1 | AAGAGAACTACCTTCCGCTGTATCG | TTCCTGACCACCATCTCCAATTCG |
| FIS1 | AATACGCCTGGTGCCTGGTTC | TAATCCCGCTGCTCCTCTTTGC |
| Mfn2 | GGAGTGGTGTGGAAGGCAGTG | GTCCAGGTCAGCCGCTCATAC |
| Mfn1 | CGTGGCAGCAGCAGAGAAGAG | ACCTCCTCCGTGACCTCCTTG |
| OPA1 | GAGGATGGTGCTGGTGGACTTG | AGGATGATGGCGTTAGGATTCTGC |
| PINK1 | CGTCTCAAAGGGAGCAGATGGG | TTAAGATGGCTTCGCTGGAGGATC |
| LC3 II | AACCAGGACAAGCAGGCAGATG | AGGCTTTCGTCTCTTCCACCATC |
| FUNDC1 | AGATAGTAATGGGTGGCGTGACTG | ACTGTGACTGGCAACCTGTAGAAG |
| Parkin | GTGGCTGGCTGTCCCAACTC | GCACTCCTCGGCACCATACTG |
| p62 | AGTTCCAGCACAGGCACAGAAG | CCACCGACTCCAAGGCTATCTTC |
| BAX | CGATGAACTGGACAACAACATGGAG | GCCACACGGAAGAAGACCTCTC |
| Cyto C | AAGGAGGCAAGCATAAGACTGGAC | ACCTTTGTTCTTGTTGGCATCTGTG |
| Bcl-2 | CGAGTGGGATACTGGAGATGAAGAC | GGTTGCTCTCAGGCTGGAAGG |
| PGC-1α | CACCAAACCCACAGAGAACAGAAAC | GTTGCGACTGCGGTTGTGTATG |
| TFAM | CCAGGAGGCTAAGGATGAGTCAG | CTGCGACGGATGAGATCACTTC |
| Nrf-1 | TGGCTGATGGAGAGGTGGAAC | GATGCTTGCGTCGTCTGGATG |
| β-Actin | CATTGTCACCAACTGGGACGATA | GGATGGCTACGTACATGGCTG |

# 4. Supplementary Reference

[1] Percie du Sert N, Hurst V, Ahluwalia A, Alam S, Avey M, Baker M, et al. The ARRIVE guidelines 2.0: Updated guidelines for reporting animal research. PLoS biology. (2020);18:e3000410.doi:10.1371/journal.pbio.3000410

[2] Manson J, Hoffman R, Chen S, Ramadan M, Billiar T. Innate-Like Lymphocytes Are Immediate Participants in the Hyper-Acute Immune Response to Trauma and Hemorrhagic Shock. Frontiers in immunology. (2019);10:1501.doi:10.3389/fimmu.2019.01501

[3] Zhang K, Jin Y, Lai D, Wang J, Wang Y, Wu X, et al. RAGE-induced ILC2 expansion in acute lung injury due to haemorrhagic shock. Thorax. (2020);75:209-19.doi:10.1136/thoraxjnl-2019-213613

[4] Xiang H, Tao Y, Jiang Z, Huang X, Wang H, Cao W, et al. Vps33B controls Treg cell suppressive function through inhibiting lysosomal nutrient sensing complex-mediated mTORC1 activation. Cell Rep. (2022);39:110943.doi:10.1016/j.celrep.2022.110943

[5] Deamer D, Akeson M, Branton D. Three decades of nanopore sequencing. Nature biotechnology. (2016);34:518-24.doi:10.1038/nbt.3423

[6] Huang Q, Su H, Qi B, Wang Y, Yan K, Wang X, et al. A SIRT1 Activator, Ginsenoside Rc, Promotes Energy Metabolism in Cardiomyocytes and Neurons. Journal of the American Chemical Society. (2021);143:1416-27.doi:10.1021/jacs.0c10836
